# Supplementary material for: Beekeepers’ perceptions toward a new omics tool for monitoring bee health in Europe
Source: PLoS One. 2025 Jan 14;20(1):e0316609. doi: 10.1371/journal.pone.0316609 (PMC11731711; doi:10.1371/journal.pone.0316609)
Supplement: S1 Appendix — (DOCX) [file pone.0316609.s001.docx]

**Supplementary materials: Beekeepers’ perceptions toward a new omics tool for monitoring bee health in Europe**

Elena Cini^1,2^*, Simon G. Potts^1^, Deepa Senapathi^1^, Matthias Albrecht^3^, Karim Arafah^4^, Dalel Askri^4^, Michel Bocquet^5^, Philippe Bulet^6^, Cecilia Costa^7^, Pilar De la Rúa^8^, Alexandra-Maria Klein^9^, Anina Knauer^3^, Marika Mänd^10^, Risto Raimets^10^, Oliver Schweiger^11,12^, Jane C. Stout^13^, Tom D. Breeze^1^*

^1^Centre for Agri-Environmental Research, School of Agriculture, Policy and Development, University of Reading, Reading, England, United Kingdom

^2^School of Environmental and Natural Sciences, Bangor University, Bangor, Wales, United Kingdom

^3^Agroecology and Environment, Agroscope, Zurich, Switzerland

^4^Plateforme BioPark d’Archamps, Archamps, France

^5^Apimedia, Pringy, Annecy, France

^6^Institute for Advanced Biosciences, CR Inserm U1209, CNRS UMR5309, Université Grenoble Alpes. Team-Verdel: ARN, Epigénétique et Stress/RNA, Epigenetics and Stress, Grenoble, France

^7^CREA Research Centre for Agriculture and Environment, Bologna, Italy

^8^Department of Zoology and Physical Anthropology, Faculty of Veterinary, University of Murcia, Murcia, Spain

^9^Chair of Nature Conservation and Landscape Ecology, University of Freiburg, Freiburg, Germany

^10^Institute of Agricultural and Environmental Sciences, Estonian University of Life Sciences, Tartu, Estonia

^11^UFZ – Helmholtz Centre for Environmental Research, Department of Community Ecology, Halle, Germany

^12^German Centre for Integrative Biodiversity Research (iDiv) Halle-Jena-Leipzig, Deutscher, Leipzig, Germany

^13^Trinity College Dublin, School of Natural Sciences, Botany Department, College Green, Dublin, Ireland

*Corresponding authors

Emails: [elena.cini.ec@gmail.com](mailto:elena.cini.ec@gmail.com) (EC), [t.d.breeze@reading.ac.uk](mailto:t.d.breeze@reading.ac.uk) (TB)

**S1 Appendix. Questionnaire survey questions and distribution**

The survey was peer reviewed by expert researchers and/or beekeepers from each country involved in the study. These experts were asked to suggest any additional answer to include in closed questions, giving any further opinion on whether a question was useful to include or not, and ensuring all questions were clear. Moreover, BIOP (BioPark Archamps) and CNRS (Centre National de la Recherche Scientifique) researchers, who are leading the production of the Bee Health Card, made sure that the tool description presented in the survey was easily comprehended and included all important elements that were provided.

| Table A. Survey questions. | |
| --- | --- |
| Question n° | **Extended question** |
| Q1 | How many years have you been practicing beekeeping?   - As hobby - As profession |
| Q2 | How many hives have you kept in the last 3 years? Please indicate the average number per year (open answer) |
| Q3 | Why do you practice beekeeping? Please tick all the options that apply.   - Awareness of threats to pollinators - Environmental concerns - Personal hobby - Providing paid pollination services to growers - Selling honey, beeswax, pollen, other products - Others (please specify) |
| Q4_1 | Are you a member of any beekeeping associations?   - Yes - No |
| Q4_2 | Please name the associations (open question). |
| Q5 | In a typical year, how often do you undertake a detailed check on your hives for each of the following health issues? (Options: Weekly, fortnightly, monthly, more than once a year, yearly, only with a reasonable suspicion, never)   - Diseases - Parasites - Nutrition - Chemical exposure |
| Q6 | Please indicate what equipment and methods of hive inspection you use to monitor the issues below. If you do not use any, please skip this question (open question).   - Diseases - Parasites - Nutrition - Chemical exposure |
| Q7 | Do you have any regular communication with growers?   - Frequent (more than twice a year) - Infrequent (once or twice a year) - I am a grower myself and manage my own hives - I do not communicate with growers |
| Q8 | How important to you are the following sources of information on beehive health? If you like, please also add the source names in the blank spaces below (options: extremely important, very important, moderately important, slightly important, not at all important).   - Scientific journals - Beekeeping associations - National bee health agencies - Newspapers - Television/radio - Social media - Online training courses - Training courses in person - Other beekeepers - NGOs - Other (please specify) |
| Q9 | In your opinion, what are the reasons for the decline of bees? (Options: strongly agree, agree, neither agree nor disagree, disagree, strongly disagree).   - The loss of natural habitats (floral and nesting resources) - The competition between managed and wild pollinators - Diseases - Parasites - Predators - Climate change - Agrochemicals - Genetic factors - Non-optimal beekeeping practices |
| Q10 | In your opinion, what are the actions to take to reduce the decline of bees? (Options: strongly agree, agree, neither agree nor disagree, disagree, strongly disagree).   - Collaborate and exchange information with growers - Choose hives location carefully - Create or manage natural habitats and flower areas - Monitor diseases - Monitor parasites - Monitor nutritional stress - Monitor exposure to agrochemicals - Optimal beekeeping practices |
| Q11 | In your opinion, what are the reasons to protect the health of bees? (Options: strongly agree, agree, neither agree nor disagree, disagree, strongly disagree).   - Economic (*e.g.* pollination contracts, income, etc.) - Legal (*e.g.* national requirements) - The perceptions of the public - The conservation of pollinators - The safety of consumers - The security of food supplies - The growth of different varieties of crops |
| Q12 | If the Bee Health Card tool was commercially available, how confident would you be that it would be effective?   - Extremely confident - Very confident - Moderately confident - Slightly confident - Not at all confident |
| Q13 | In your opinion, what could be the barriers to using the Bee Health Card tool? (Options: strongly agree, agree, neither agree nor disagree, disagree, strongly disagree).   - Poor communication with growers - The cost of it - I am not sure it is effective - It seems time-consuming - It seems difficult to use - I am not aware of the importance of using it |
| Q14 | In your opinion, what could be the benefits to you to using the Bee Health Card tool? (Options: strongly agree, agree, neither agree nor disagree, disagree, strongly disagree).   - Better communication with growers - It helps increase productivity - It seems quick and easy to use - It reduces treatment costs - It enhances crop pollination - It increases the health of bee colonies - It helps protect the environment - It helps protect pollinators |
| Q15 | If the Bee Health Card tool was demonstrated to diagnose colony health issues efficiently and improve the colony performance, would you be interested in using it with economic incentives (*e.g.* subsidies, grants, certified products, etc.)?   - Yes, even with extra costs to me - Yes, only if there were no extra costs to me - No |
| Q16 | If the Bee Health Card tool was demonstrated to diagnose colony health issues efficiently and improve the colony performance, would you be interested in using it without economic incentives (*i.e.* no subsidies, grants, certified products, etc.)?   - Yes, even with extra costs to me - Yes, only if there were no extra costs to me - No |
| Q17 | Considering the expected benefits and cost, how many times in a typical year would you use the Bee Health Card tool with economic incentives (*e.g.* subsidies, grants, certified products, etc.)?   - Regularly (at least once a month) - Irregularly (a few times a year) - Only with a reasonable suspicion - Never   (Q17 was only shown to respondents who stated to be interested in using the Bee Health Card with economic incentives) |
| Q18 | Considering the expected benefits and cost, how many times in a typical year would you use the Bee Health Card tool without economic incentives (*i.e.* no subsidies, grants, certified products, etc.)?   - Regularly (at least once a month) - Irregularly (a few times a year) - Only with a reasonable suspicion - Never   (Q18 was only shown to respondents who stated to be interested in using the Bee Health Card without economic incentives) |
| Q19 | In your opinion, are there any specific health issues that you would like the Bee Health Card tool to be able to detect in your colonies? (Open question) |

| Table B. Survey languages of distribution. | |
| --- | --- |
| Country | **Survey language** |
| Estonia | Estonian |
| Germany | German |
| Ireland | English |
| Italy | Italian |
| Spain | Spanish |
| Switzerland | German |
| UK | English |

| Table C. Channels used to advertise the survey. A reminder to ask researchers to further advertise the survey was sent on October 24th, 2020. | |
| --- | --- |
| Country | **Advertisement channels** |
| Estonia | Local Estonian beekeeping associations |
| Germany | Local German beekeeping associations |
| Ireland | ‘FIBKA’ Facebook page and Sept 2020 newsletter |
|  | ‘NIHBS’ Aug 2020 News Update |
|  | ‘Beekeepers of Ireland’ Facebook page |
|  | ‘Cork Beekeepers’ Facebook page |
|  | Twitter account of lead researcher for Ireland |
| Italy | ‘UNAAPI’ Facebook page |
| Spain | Twitter and Facebook accounts of lead researcher for Spain |
|  | ‘ADEA-ASAJA’ contact list and Twitter account |
| Switzerland | Local Swiss beekeeping associations |
| UK | ‘BBKA’ Facebook and Twitter pages, website |
|  | Kent beekeepers involved in the PoshBee project |
|  | ‘Barnsley BKA’, circulated to members |
|  | ‘Mid Bucks BKA’ Aug 2020 newsletter |
|  | ‘Winchester BKA’ Aug 2020 newsletter |
|  | ‘Bee Craft Magazine’ Sept 2020 issue |
|  | ‘Rustley BKA’, circulated to members |
|  | Twitter and Facebook accounts of lead researcher for the UK |
| Other sources | |
| Pensoft | PoshBee Twitter, Facebook, website |
