# Supplementary material for: Beekeepers’ perceptions toward a new omics tool for monitoring bee health in Europe
Source: PLoS One. 2025 Jan 14;20(1):e0316609. doi: 10.1371/journal.pone.0316609 (PMC11731711; doi:10.1371/journal.pone.0316609)
Supplement: S2 Appendix — (DOCX) [file pone.0316609.s002.docx]

**Supplementary materials: Beekeepers’ perceptions toward a new omics tool for monitoring bee health in Europe**

Elena Cini^1,2^*, Simon G. Potts^1^, Deepa Senapathi^1^, Matthias Albrecht^3^, Karim Arafah^4^, Dalel Askri^4^, Michel Bocquet^5^, Philippe Bulet^6^, Cecilia Costa^7^, Pilar De la Rúa^8^, Alexandra-Maria Klein^9^, Anina Knauer^3^, Marika Mänd^10^, Risto Raimets^10^, Oliver Schweiger^11,12^, Jane C. Stout^13^, Tom D. Breeze^1^*

^1^Centre for Agri-Environmental Research, School of Agriculture, Policy and Development, University of Reading, Reading, England, United Kingdom

^2^School of Environmental and Natural Sciences, Bangor University, Bangor, Wales, United Kingdom

^3^Agroecology and Environment, Agroscope, Zurich, Switzerland

^4^Plateforme BioPark d’Archamps, Archamps, France

^5^Apimedia, Pringy, Annecy, France

^6^Institute for Advanced Biosciences, CR Inserm U1209, CNRS UMR5309, Université Grenoble Alpes. Team-Verdel: ARN, Epigénétique et Stress/RNA, Epigenetics and Stress, Grenoble, France

^7^CREA Research Centre for Agriculture and Environment, Bologna, Italy

^8^Department of Zoology and Physical Anthropology, Faculty of Veterinary, University of Murcia, Murcia, Spain

^9^Chair of Nature Conservation and Landscape Ecology, University of Freiburg, Freiburg, Germany

^10^Institute of Agricultural and Environmental Sciences, Estonian University of Life Sciences, Tartu, Estonia

^11^UFZ – Helmholtz Centre for Environmental Research, Department of Community Ecology, Halle, Germany

^12^German Centre for Integrative Biodiversity Research (iDiv) Halle-Jena-Leipzig, Deutscher, Leipzig, Germany

^13^Trinity College Dublin, School of Natural Sciences, Botany Department, College Green, Dublin, Ireland

*Corresponding authors

Emails: [elena.cini.ec@gmail.com](mailto:elena.cini.ec@gmail.com) (EC), [t.d.breeze@reading.ac.uk](mailto:t.d.breeze@reading.ac.uk) (TB)

**S2 Appendix. Materials required for the Bee Health Card**

| Table A. Materials required for haemolymph collection and postage. | | | | |
| --- | --- | --- | --- | --- |
| Item | **Quantity/sample** | **Cost** | **Cost/Sample** | **Source** |
| Silicon collector tube | 0.8 | € 1.80 | € 1.44 | <https://www.fishersci.co.uk/shop/products/silicone-tubes-4/10430313#silicon%20tube> |
| Filter tip 1000 μL | 5 | € 0.15 | € 0.75 | <https://www.fishersci.co.uk/shop/products/thermo-scientific-art-barrier-reload-insert-pipette-tips-10/12637696#200ul%20tip> |
| Capillary holder | 1 | € 2.00 | € 2.00 | <https://www.fishersci.co.uk/shop/products/adapter-quiksip-pvc-for-capillaries/10479862#?keyword=Capillary%20pipete> |
| Bee holder | 0.06 | € 1.80 | € 0.11 | <https://www.fishersci.co.uk/shop/products/silicone-tubes-4/10430313#silicon%20tube> |
| Total reusable |  |  | **€ 4.30** |  |
| Capillaries (80mm) | 0.5 | € 0.08 | € 0.042 | <https://www.fishersci.co.uk/shop/products/capillary-tube-2/10309901#Capillary> |
| Coated microcentrifuge tubes 1.5 mL | 10 | € 0.03 | € 0.293 | <https://www.fishersci.co.uk/shop/products/fisherbrand-premium-microcentrifuge-tubes-1-5ml/11926955#coated%20microcentrifuge%20tubes%201.5ml> |
| Freezer Gel packs | 2 | € 0.82 | € 1.64 | <https://www.fishersci.co.uk/shop/products/sonoco-thermosafe-polarpack-gel-packs-18/13009342#freezer%20gel%20pack> |
| Packaging | 1 | € 0.27 | € 0.27 | <https://www.amazon.co.uk/Gold-Bubble-Padded-Envelopes-240x320mm/dp/B004K26SO6> |
| Total per use |  |  | **€ 2.24** |  |
| Postage (Estonia) | 1 | € 26.00 | € 26.00 | Potts et al. (2021) – No international carrier is currently undertaking domestic deliveries |
| Postage (Germany) | 1 | € 32.03 | € 32.03 | <https://mydhl.express.dhl/de/en/shipment.html#/rate-and-quote#address-details> and <https://www.tnt.com/express/de_de/site/home/applications/obt.html?respCountry=de&respLang=de&origincountry=DE&navigation=1&destcountry=DE> |
| Postage (Ireland) | 1 | € 25.69 | € 25.69 | [https://mydhl.express.dhl/ie/en/shipment.html#/rate-and-quote#delivery-options](https://mydhl.express.dhl/ie/en/shipment.html#/rate-and-quote) <https://www.tnt.com/express/en_ie/site/get-quote.html> |
| Postage (Italy) | 1 | € 20.32 | € 20.32 | <https://www.tnt.com/express/it_it/site/home/Spedisci-ora.html?source=legacy_obt>  [https://mydhl.express.dhl/it/en/shipment.html#/rate-and-quote#delivery-options](https://mydhl.express.dhl/it/en/shipment.html#/rate-and-quote) |
| Postage (Spain) | 1 | € 18.74 | € 18.74 | <https://www.tnt.com/express/es_es/site/obtener-presupuesto.html> <https://mydhl.express.dhl/es/en/shipment.html#/rate-and-quote> |
| Postage (Switzerland) | 1 | € 21.79 | € 21.79 | <https://www.dhl.com/ch-en/home/get-a-quote.html> |
| Postage (UK) | 1 | € 8.95 | € 8.95 | <https://direct.tnt.co.uk/quick-quote>  <https://www.dhl.com/gb-en/home/get-a-quote.html> |
| Average |  |  | **€ 16.02** |  |

| Table B. Consumable materials, time and data required for lab analysis. | | | | |
| --- | --- | --- | --- | --- |
| Item | **Units/sample** | **Cost/1 unit (l, kg, or day)** | **Unit/Sample** | **Source** |
| Eppendorf tubes (0.5ml) | 1 | € 0.10 | € 0.104 | <https://www.fishersci.co.uk/shop/products/eppendorf-0-5ml-pcr-tubes/10401203#?keyword=Eppendorf%200.5ml%20tube> |
| Adapted pipette tips (10μL) | 1 | € 0.10 | € 0.099 | <https://www.fishersci.co.uk/shop/products/axygen-10-l-microvolume-tips-19/12756749> |
| Adapted pipette tips (200μL) | 1 | € 0.15 | € 0.153 | <https://www.fishersci.co.uk/shop/products/art-softfit-l-barrier-hinged-rack-pipette-tips/11585454#200ul%20tip> |
| Adapted pipette tips (1000 μL) | 1 | € 0.15 | € 0.151 | <https://www.fishersci.co.uk/shop/products/thermo-scientific-art-barrier-reload-insert-pipette-tips-10/12637696#200ul%20tip> |
| Kimwipes® disposable wipers | 1 | € 0.04 | € 0.036 | <https://www.fishersci.co.uk/shop/products/kimwipes-delicate-task-wipers-2/13258179#?keyword=Kimwipes%C2%AE> |
| 15 mL Falcon tubes | 0.5 | € 0.32 | € 0.160 | <https://www.fishersci.co.uk/shop/products/falcon-15ml-conical-centrifuge-tubes-5/10468502> |
| 50 mL Falcon tubes | 0.5 | € 0.35 | € 0.176 | <https://www.fishersci.co.uk/shop/products/falcon-50ml-conical-centrifuge-tubes-2/12716688#Falcon%2050%20mL%20Conical%20Centrifuge%20Tubes> |
| MALDI matrix | 0.0078125 | € 86.51 | € 0.676 | <https://www.fishersci.co.uk/shop/products/alpha-cyano-4-%20hydroxycinnamic-acid-ultrapure-maldi-matrix-thermo-scientific/15410777#?keyword=A-Cyano-4-hydroxycinnamic%20acid> |
| Total |  |  | **1.554 €** |  |
| Data storage (1gb) - calibration | 0.0006 | € 0.02 | <€ 0.001 | <https://cloud.google.com/storage/pricing#europe> |
| Data storage (1gb) - samples | 0.0017 | € 0.02 | <€ 0.001 | <https://cloud.google.com/storage/pricing#europe> |
| Staff days (Estonia) | 0.005 | € 133.72 | € 0.67 | Potts et al. (2021) – No international carrier is currently undertaking domestic deliveries |
| Staff days (Germany) | 0.005 | € 237.27 | € 1.19 | Potts et al. (2021) – No international carrier is currently undertaking domestic deliveries |
| Staff days (Ireland) | 0.005 | € 212.14 | € 1.06 | Potts et al. (2021) – No international carrier is currently undertaking domestic deliveries |
| Staff days (Italy) | 0.005 | € 227.51 | € 1.14 | Potts et al. (2021) – No international carrier is currently undertaking domestic deliveries |
| Staff days (Spain) | 0.005 | € 195.65 | € 0.98 | Potts et al. (2021) – No international carrier is currently undertaking domestic deliveries |
| Staff days (Switzerland) | 0.005 | € 299.32 | € 1.50 | Potts et al. (2021) – No international carrier is currently undertaking domestic deliveries |
| Staff days (UK) | 0.005 | € 299.32 | € 1.50 | Potts et al. (2021) – No international carrier is currently undertaking domestic deliveries |

| Table C. Additional lab materials. | |
| --- | --- |
| MALDI equipment | Software (acquisition, post-processing), database of reference spectra, and all the devises required for the functionality of the mass spectrometer (Lab. Environment), a kit for sample traceability by barcoding |
| General equipment | Centrifuge for Eppendorf tubes (refrigerated is better but not mandatory), ice machine to keep sample sin the cold, a refrigerator and a freezer (-20°C being sufficient), a glass developing chamber for TLC adapted to the size of the reusable MALDI plates |
| Chemicals | Ultrapure water, acetonitrile, ethanol, methanol, acetone, trifluoracetic acid, phenylmethanesulfonylfluoride (PMSF, protease inhibitor), phenylthiourea (PTU, inhibitor of melanisation), MALDI matrix (4HCCA, A-Cyano-4-hydroxycinnamic acid preferentially), peptide/protein kits for equipment calibration. We developed a specific calibration kit for MALDI BeeTyping® |

| Table D. Administration costs. Source: Potts et al., 2021. | | |
| --- | --- | --- |
| Country | **Admin cost/year** | **Notes** |
| Estonia | € 29,419.20 |  |
| Germany | € 61,300.00 |  |
| Ireland | € 53,337.00 |  |
| Spain | € 48,851.76 |  |
| Switzerland | € 40,642.73 | Not included in Potts et al (2021.), thus costs were assumed to be the same as the UK. |
| United Kingdom | € 40,642.73 |  |
