# Supplementary material for: Beekeepers’ perceptions toward a new omics tool for monitoring bee health in Europe
Source: PLoS One. 2025 Jan 14;20(1):e0316609. doi: 10.1371/journal.pone.0316609 (PMC11731711; doi:10.1371/journal.pone.0316609)
Supplement: S3 Appendix — (DOCX) [file pone.0316609.s003.docx]

**Supplementary materials: Beekeepers’ perceptions toward a new omics tool for monitoring bee health in Europe**

Elena Cini^1,2^*, Simon G. Potts^1^, Deepa Senapathi^1^, Matthias Albrecht^3^, Karim Arafah^4^, Dalel Askri^4^, Michel Bocquet^5^, Philippe Bulet^6^, Cecilia Costa^7^, Pilar De la Rúa^8^, Alexandra-Maria Klein^9^, Anina Knauer^3^, Marika Mänd^10^, Risto Raimets^10^, Oliver Schweiger^11,12^, Jane C. Stout^13^, Tom D. Breeze^1^*

^1^Centre for Agri-Environmental Research, School of Agriculture, Policy and Development, University of Reading, Reading, England, United Kingdom

^2^School of Environmental and Natural Sciences, Bangor University, Bangor, Wales, United Kingdom

^3^Agroecology and Environment, Agroscope, Zurich, Switzerland

^4^Plateforme BioPark d’Archamps, Archamps, France

^5^Apimedia, Pringy, Annecy, France

^6^Institute for Advanced Biosciences, CR Inserm U1209, CNRS UMR5309, Université Grenoble Alpes. Team-Verdel: ARN, Epigénétique et Stress/RNA, Epigenetics and Stress, Grenoble, France

^7^CREA Research Centre for Agriculture and Environment, Bologna, Italy

^8^Department of Zoology and Physical Anthropology, Faculty of Veterinary, University of Murcia, Murcia, Spain

^9^Chair of Nature Conservation and Landscape Ecology, University of Freiburg, Freiburg, Germany

^10^Institute of Agricultural and Environmental Sciences, Estonian University of Life Sciences, Tartu, Estonia

^11^UFZ – Helmholtz Centre for Environmental Research, Department of Community Ecology, Halle, Germany

^12^German Centre for Integrative Biodiversity Research (iDiv) Halle-Jena-Leipzig, Deutscher, Leipzig, Germany

^13^Trinity College Dublin, School of Natural Sciences, Botany Department, College Green, Dublin, Ireland

*Corresponding authors

Emails: [elena.cini.ec@gmail.com](mailto:elena.cini.ec@gmail.com) (EC), [t.d.breeze@reading.ac.uk](mailto:t.d.breeze@reading.ac.uk) (TB)

**S3 Appendix. Survey results**

**Sample description**

| Table A. Average number of beehives kept by each respondent in the last 3 years. The number varies across countries, with the majority of beekeepers in Italy having the highest average (50/year), and Ireland and the UK the lowest (3/year). This may be explained by the fact that the majority of participants based in Ireland and UK are hobbyist, while those based in Italy are mainly professionals (see table ‘Hobbyist vs. professional beekeepers’ below). | | | | | | | | | | | | | | | | | |
| --- | --- | --- | --- | --- | --- | --- | --- | --- | --- | --- | --- | --- | --- | --- | --- | --- | --- |
| Average n° per year | **Estonia** | | **Germany** | | **Ireland** | | **Italy** | | **Spain** | | **Switzerland** | | **UK** | | **Total** | |  |
|  | **n** | **%** | **n** | **%** | **n** | **%** | **n** | **%** | **n** | **%** | **n** | **%** | **n** | **%** | **n** | **%** |  |
| 0 | 0 | 0.00 | 1 | 3.03 | 0 | 0.00 | 0 | 0.00 | 0 | 0.00 | 0 | 0.00 | 0 | 0.00 | 1 | 0.21 |  |
| 1 | 1 | 3.13 | 1 | 3.03 | 12 | 10.43 | 1 | 1.52 | 0 | 0.00 | 0 | 0.00 | 5 | 3.68 | 20 | 4.22 |  |
| 1.5 | 0 | 0.00 | 0 | 0.00 | 1 | 0.87 | 0 | 0.00 | 0 | 0.00 | 0 | 0.00 | 0 | 0.00 | 1 | 0.21 |  |
| 2 | 0 | 0.00 | 2 | 6.06 | 19 | 16.52 | 0 | 0.00 | 0 | 0.00 | 0 | 0.00 | 18 | 13.24 | 39 | 8.23 |  |
| 2.5 | 0 | 0.00 | 1 | 3.03 | 0 | 0.00 | 0 | 0.00 | 0 | 0.00 | 0 | 0.00 | 0 | 0.00 | 1 | 0.21 |  |
| 3 | 1 | 3.13 | 1 | 3.03 | 22 | 19.13 | 3 | 4.55 | 0 | 0.00 | 1 | 1.92 | 29 | 21.32 | 57 | 12.03 |  |
| 3.5 | 0 | 0.00 | 0 | 0.00 | 1 | 0.87 | 0 | 0.00 | 0 | 0.00 | 0 | 0.00 | 0 | 0.00 | 1 | 0.21 |  |
| 4 | 1 | 3.13 | 1 | 3.03 | 9 | 7.83 | 2 | 3.03 | 0 | 0.00 | 2 | 3.85 | 17 | 12.50 | 32 | 6.75 |  |
| 5 | 2 | 6.25 | 4 | 12.12 | 7 | 6.09 | 3 | 4.55 | 1 | 2.50 | 1 | 1.92 | 12 | 8.82 | 30 | 6.33 |  |
| 6 | 0 | 0.00 | 5 | 15.15 | 5 | 4.35 | 3 | 4.55 | 0 | 0.00 | 2 | 3.85 | 12 | 8.82 | 27 | 5.70 |  |
| 7 | 0 | 0.00 | 1 | 3.03 | 5 | 4.35 | 1 | 1.52 | 1 | 2.50 | 1 | 1.92 | 2 | 1.47 | 11 | 2.32 |  |
| 8 | 1 | 3.13 | 0 | 0.00 | 1 | 0.87 | 0 | 0.00 | 1 | 2.50 | 1 | 1.92 | 6 | 4.41 | 10 | 2.11 |  |
| 8,5 | 0 | 0.00 | 0 | 0.00 | 0 | 0.00 | 0 | 0.00 | 0 | 0.00 | 0 | 0.00 | 1 | 0.74 | 1 | 0.21 |  |
| 9 | 0 | 0.00 | 0 | 0.00 | 0 | 0.00 | 1 | 1.52 | 1 | 2.50 | 0 | 0.00 | 0 | 0.00 | 2 | 0.42 |  |
| 10 | 4 | 12.50 | 2 | 6.06 | 9 | 7.83 | 3 | 4.55 | 3 | 7.50 | 7 | 13.46 | 9 | 6.62 | 37 | 7.81 |  |
| 11 | 0 | 0.00 | 0 | 0.00 | 1 | 0.87 | 1 | 1.52 | 0 | 0.00 | 0 | 0.00 | 0 | 0.00 | 2 | 0.42 |  |
| 12 | 0 | 0.00 | 3 | 9.09 | 3 | 2.61 | 1 | 1.52 | 1 | 2.50 | 3 | 5.77 | 1 | 0.74 | 12 | 2.53 |  |
| 13 | 0 | 0.00 | 0 | 0.00 | 0 | 0.00 | 1 | 1.52 | 0 | 0.00 | 0 | 0.00 | 0 | 0.00 | 1 | 0.21 |  |
| 14 | 1 | 3.13 | 1 | 3.03 | 2 | 1.74 | 0 | 0.00 | 1 | 2.50 | 1 | 1.92 | 0 | 0.00 | 6 | 1.27 |  |
| 15 | 0 | 0.00 | 3 | 9.09 | 3 | 2.61 | 3 | 4.55 | 5 | 12.50 | 1 | 1.92 | 1 | 0.74 | 16 | 3.38 |  |
| 16 | 0 | 0.00 | 2 | 6.06 | 0 | 0.00 | 1 | 1.52 | 0 | 0.00 | 1 | 1.92 | 2 | 1.47 | 6 | 1.27 |  |
| 16.5 | 1 | 3.13 | 0 | 0.00 | 0 | 0.00 | 0 | 0.00 | 0 | 0.00 | 0 | 0.00 | 0 | 0.00 | 1 | 0.21 |  |
| 17 | 0 | 0.00 | 0 | 0.00 | 0 | 0.00 | 1 | 1.52 | 0 | 0.00 | 0 | 0.00 | 0 | 0.00 | 1 | 0.21 |  |
| 17.5 | 0 | 0.00 | 0 | 0.00 | 0 | 0.00 | 0 | 0.00 | 1 | 2.50 | 0 | 0.00 | 0 | 0.00 | 1 | 0.21 |  |
| 18 | 0 | 0.00 | 0 | 0.00 | 0 | 0.00 | 2 | 3.03 | 0 | 0.00 | 3 | 5.77 | 1 | 0.74 | 6 | 1.27 |  |
| 19 | 0 | 0.00 | 0 | 0.00 | 2 | 1.74 | 0 | 0.00 | 0 | 0.00 | 0 | 0.00 | 0 | 0.00 | 2 | 0.42 |  |
| 20 | 0 | 0.00 | 1 | 3.03 | 3 | 2.61 | 3 | 4.55 | 1 | 2.50 | 7 | 13.46 | 4 | 2.94 | 19 | 4.01 |  |
| 22 | 0 | 0.00 | 0 | 0.00 | 0 | 0.00 | 0 | 0.00 | 0 | 0.00 | 1 | 1.92 | 0 | 0.00 | 1 | 0.21 |  |
| 24 | 0 | 0.00 | 1 | 3.03 | 0 | 0.00 | 0 | 0.00 | 0 | 0.00 | 0 | 0.00 | 0 | 0.00 | 1 | 0.21 |  |
| 25 | 2 | 6.25 | 0 | 0.00 | 0 | 0.00 | 4 | 6.06 | 1 | 2.50 | 4 | 7.69 | 4 | 2.94 | 15 | 3.16 |  |
| 30 | 1 | 3.13 | 0 | 0.00 | 2 | 1.74 | 0 | 0.00 | 5 | 12.50 | 7 | 13.46 | 3 | 2.21 | 18 | 3.80 |  |
| 32 | 1 | 3.13 | 0 | 0.00 | 0 | 0.00 | 0 | 0.00 | 0 | 0.00 | 1 | 1.92 | 0 | 0.00 | 2 | 0.42 |  |
| 35 | 0 | 0.00 | 0 | 0.00 | 0 | 0.00 | 0 | 0.00 | 0 | 0.00 | 2 | 3.85 | 1 | 0.74 | 3 | 0.63 |  |
| 40 | 0 | 0.00 | 1 | 3.03 | 2 | 1.74 | 1 | 1.52 | 2 | 5.00 | 2 | 3.85 | 1 | 0.74 | 9 | 1.90 |  |
| 45 | 2 | 6.25 | 0 | 0.00 | 0 | 0.00 | 1 | 1.52 | 1 | 2.50 | 0 | 0.00 | 0 | 0.00 | 4 | 0.84 |  |
| 50 | 1 | 3.13 | 1 | 3.03 | 0 | 0.00 | 7 | 10.61 | 2 | 5.00 | 3 | 5.77 | 1 | 0.74 | 15 | 3.16 |  |
| 54 | 0 | 0.00 | 0 | 0.00 | 0 | 0.00 | 0 | 0.00 | 0 | 0.00 | 0 | 0.00 | 1 | 0.74 | 1 | 0.21 |  |
| 60 | 0 | 0.00 | 0 | 0.00 | 1 | 0.87 | 1 | 1.52 | 0 | 0.00 | 0 | 0.00 | 0 | 0.00 | 2 | 0.42 |  |
| 65 | 0 | 0.00 | 0 | 0.00 | 1 | 0.87 | 0 | 0.00 | 0 | 0.00 | 0 | 0.00 | 0 | 0.00 | 1 | 0.21 |  |
| 70 | 1 | 3.13 | 0 | 0.00 | 0 | 0.00 | 0 | 0.00 | 2 | 5.00 | 0 | 0.00 | 0 | 0.00 | 3 | 0.63 |  |
| 75 | 0 | 0.00 | 0 | 0.00 | 0 | 0.00 | 2 | 3.03 | 0 | 0.00 | 0 | 0.00 | 1 | 0.74 | 3 | 0.63 |  |
| 80 | 1 | 3.13 | 0 | 0.00 | 0 | 0.00 | 0 | 0.00 | 1 | 2.50 | 1 | 1.92 | 1 | 0.74 | 4 | 0.84 |  |
| 85 | 0 | 0.00 | 0 | 0.00 | 1 | 0.87 | 0 | 0.00 | 0 | 0.00 | 0 | 0.00 | 0 | 0.00 | 1 | 0.21 |  |
| 100 | 2 | 6.25 | 1 | 3.03 | 1 | 0.87 | 3 | 4.55 | 6 | 15.00 | 0 | 0.00 | 3 | 2.21 | 16 | 3.38 |  |
| 119 | 0 | 0.00 | 0 | 0.00 | 1 | 0.87 | 0 | 0.00 | 0 | 0.00 | 0 | 0.00 | 0 | 0.00 | 1 | 0.21 |  |
| 120 | 1 | 3.13 | 0 | 0.00 | 0 | 0.00 | 1 | 1.52 | 0 | 0.00 | 0 | 0.00 | 0 | 0.00 | 2 | 0.42 |  |
| 149 | 0 | 0.00 | 0 | 0.00 | 0 | 0.00 | 0 | 0.00 | 1 | 2.50 | 0 | 0.00 | 0 | 0.00 | 1 | 0.21 |  |
| 150 | 1 | 3.13 | 0 | 0.00 | 0 | 0.00 | 5 | 7.58 | 0 | 0.00 | 0 | 0.00 | 0 | 0.00 | 6 | 1.27 |  |
| 155 | 1 | 3.13 | 0 | 0.00 | 0 | 0.00 | 0 | 0.00 | 0 | 0.00 | 0 | 0.00 | 0 | 0.00 | 1 | 0.21 |  |
| 160 | 2 | 6.25 | 0 | 0.00 | 0 | 0.00 | 0 | 0.00 | 0 | 0.00 | 0 | 0.00 | 0 | 0.00 | 2 | 0.42 |  |
| 170 | 0 | 0.00 | 0 | 0.00 | 0 | 0.00 | 1 | 1.52 | 0 | 0.00 | 0 | 0.00 | 0 | 0.00 | 1 | 0.21 |  |
| 180 | 0 | 0.00 | 0 | 0.00 | 1 | 0.87 | 1 | 1.52 | 0 | 0.00 | 0 | 0.00 | 0 | 0.00 | 2 | 0.42 |  |
| 200 | 2 | 6.25 | 0 | 0.00 | 0 | 0.00 | 1 | 1.52 | 2 | 5.00 | 0 | 0.00 | 0 | 0.00 | 5 | 1.05 |  |
| 230 | 0 | 0.00 | 0 | 0.00 | 0 | 0.00 | 2 | 3.03 | 0 | 0.00 | 0 | 0.00 | 0 | 0.00 | 2 | 0.42 |  |
| 250 | 0 | 0.00 | 0 | 0.00 | 0 | 0.00 | 1 | 1.52 | 0 | 0.00 | 0 | 0.00 | 0 | 0.00 | 1 | 0.21 |  |
| 280 | 1 | 3.13 | 0 | 0.00 | 0 | 0.00 | 0 | 0.00 | 0 | 0.00 | 0 | 0.00 | 0 | 0.00 | 1 | 0.21 |  |
| 350 | 0 | 0.00 | 0 | 0.00 | 0 | 0.00 | 1 | 1.52 | 0 | 0.00 | 0 | 0.00 | 0 | 0.00 | 1 | 0.21 |  |
| 500 | 1 | 3.13 | 0 | 0.00 | 0 | 0.00 | 0 | 0.00 | 0 | 0.00 | 0 | 0.00 | 0 | 0.00 | 1 | 0.21 |  |
| 600 | 0 | 0.00 | 0 | 0.00 | 0 | 0.00 | 0 | 0.00 | 1 | 2.50 | 0 | 0.00 | 0 | 0.00 | 1 | 0.21 |  |
| 1000 | 0 | 0.00 | 0 | 0.00 | 0 | 0.00 | 2 | 3.03 | 0 | 0.00 | 0 | 0.00 | 0 | 0.00 | 2 | 0.42 |  |
| 1500 | 0 | 0.00 | 0 | 0.00 | 0 | 0.00 | 1 | 1.52 | 0 | 0.00 | 0 | 0.00 | 0 | 0.00 | 1 | 0.21 |  |
| N/A | 0 | 0.00 | 0 | 0.00 | 0 | 0.00 | 1 | 1.52 | 0 | 0.00 | 0 | 0.00 | 0 | 0.00 | 1 | 0.21 |  |
| Total | **32** |  | **33** |  | **115** |  | **66** |  | **40** |  | **52** |  | **136** |  | **474** |  |  |

| Table B. Percentages of hobbyist vs professional participants. Percentages were obtained from Q1 (‘How many years have you been practicing beekeeping as hobby and as profession?’). If years of profession = 0, beekeepers were counted only as hobbyists. If years of profession > 0, beekeepers were counted among professionals. The majority of recruited respondents are hobbyist. The most common answers are highlighted in bold. | | | | | | | | |
| --- | --- | --- | --- | --- | --- | --- | --- | --- |
| Respondents | **Estonia** | **Germany** | **Ireland** | **Italy** | **Spain** | **Switzerland** | **UK** | **Total** |
| Hobbyists | 43.75% | 87.88% | **89.57%** | 37.88% | **55.00%** | **82.69%** | **85.29%** | **74.26%** |
| Professionals | **50.00%** | 9.09% | 10.43% | **60.61%** | 45.00% | 11.54% | 13.97% | 24.05% |

| Table C. Participants’ years of practicing beekeeping as hobby (A) vs profession (B). | | | | | | | | | | | | | | | | | | | | | | | | | | | | | | | | | | | | | | | |
| --- | --- | --- | --- | --- | --- | --- | --- | --- | --- | --- | --- | --- | --- | --- | --- | --- | --- | --- | --- | --- | --- | --- | --- | --- | --- | --- | --- | --- | --- | --- | --- | --- | --- | --- | --- | --- | --- | --- | --- |
| A: Years as hobbyists | **Estonia** | | | | **Germany** | | | | | **Ireland** | | | | | **Italy** | | | | | **Spain** | | | | | **Switzerland** | | | | | **UK** | | | | | **Total** | | | | |
|  | **n** | | **%** | | **n** | | | **%** | | **n** | | | **%** | | **n** | | | **%** | | **n** | | | **%** | | **n** | | | **%** | | **n** | **%** | | | | **n** | | | **%** | |
| 0 | 5 | | 15.63 | | 1 | | | 3.13 | | 1 | | | 0.87 | | 7 | | | 10.61 | | 1 | | | 2.50 | | 0 | | | 0.00 | | 0 | 0.00 | | | | 15 | | | 3.18 | |
| 0.3 | 0 | | 0.00 | | 0 | | | 0.00 | | 0 | | | 0.00 | | 0 | | | 0.00 | | 0 | | | 0.00 | | 0 | | | 0.00 | | 1 | 0.74 | | | | 1 | | | 0.21 | |
| 0.5 | 0 | | 0.00 | | 0 | | | 0.00 | | 1 | | | 0.87 | | 0 | | | 0.00 | | 0 | | | 0.00 | | 0 | | | 0.00 | | 1 | 0.74 | | | | 2 | | | 0.42 | |
| 1 | 1 | | 3.13 | | 1 | | | 3.13 | | 12 | | | 10.43 | | 6 | | | 9.09 | | 2 | | | 5.00 | | 0 | | | 0.00 | | 10 | 7.41 | | | | 32 | | | 6.78 | |
| 1.5 | 0 | | 0.00 | | 0 | | | 0.00 | | 1 | | | 0.87 | | 0 | | | 0.00 | | 0 | | | 0.00 | | 0 | | | 0.00 | | 0 | 0.00 | | | | 1 | | | 0.21 | |
| 2 | 4 | | 12.50 | | 1 | | | 3.13 | | 12 | | | 10.43 | | 8 | | | 12.12 | | 3 | | | 7.50 | | 0 | | | 0.00 | | 16 | 11.85 | | | | 44 | | | 9.32 | |
| 3 | 0 | | 0.00 | | 5 | | | 15.62 | | 11 | | | 9.57 | | 6 | | | 9.09 | | 2 | | | 5.00 | | 2 | | | 3.85 | | 8 | 5.93 | | | | 34 | | | 7.20 | |
| 4 | 1 | | 3.13 | | 2 | | | 6.25 | | 20 | | | 17.39 | | 8 | | | 12.12 | | 1 | | | 2.50 | | 2 | | | 3.85 | | 14 | 10.37 | | | | 48 | | | 10.1 | |
| 5 | 4 | | 12.50 | | 0 | | | 0.00 | | 6 | | | 5.22 | | 13 | | | 19.70 | | 5 | | | 12.50 | | 4 | | | 7.69 | | 12 | 8.89 | | | | 44 | | | 9.32 | |
| 6 | 4 | | 12.50 | | 3 | | | 9.38 | | 4 | | | 3.48 | | 4 | | | 6.06 | | 2 | | | 5.00 | | 2 | | | 3.85 | | 10 | 7.41 | | | | 29 | | | 6.14 | |
| 7 | 1 | | 3.13 | | 1 | | | 3.13 | | 5 | | | 4.35 | | 2 | | | 3.03 | | 4 | | | 10.00 | | 2 | | | 3.85 | | 6 | 4.44 | | | | 21 | | | 4.45 | |
| 8 | 2 | | 6.25 | | 2 | | | 6.25 | | 6 | | | 5.22 | | 1 | | | 1.52 | | 1 | | | 2.50 | | 3 | | | 5.77 | | 3 | 2.22 | | | | 18 | | | 3.81 | |
| 9 | 0 | | 0.00 | | 1 | | | 3.13 | | 4 | | | 3.48 | | 1 | | | 1.52 | | 0 | | | 0.00 | | 3 | | | 5.77 | | 3 | 2.22 | | | | 12 | | | 2.54 | |
| 10 | 2 | | 6.25 | | 1 | | | 3.13 | | 11 | | | 9.57 | | 0 | | | 0.00 | | 0 | | | 0.00 | | 5 | | | 9.62 | | 11 | 8.15 | | | | 30 | | | 6.36 | |
| 11 | 0 | | 0.00 | | 2 | | | 6.25 | | 0 | | | 0.00 | | 0 | | | 0.00 | | 0 | | | 0.00 | | 1 | | | 1.92 | | 3 | 2.22 | | | | 6 | | | 1.27 | |
| 12 | 0 | | 0.00 | | 0 | | | 0.00 | | 2 | | | 1.74 | | 1 | | | 1.52 | | 1 | | | 2.50 | | 2 | | | 3.85 | | 4 | 2.96 | | | | 10 | | | 2.12 | |
| 13 | 1 | | 3.13 | | 2 | | | 6.25 | | 2 | | | 1.74 | | 1 | | | 1.52 | | 0 | | | 0.00 | | 2 | | | 3.85 | | 1 | 0.74 | | | | 9 | | | 1.91 | |
| 14 | 0 | | 0.00 | | 1 | | | 3.13 | | 2 | | | 1.74 | | 0 | | | 0.00 | | 0 | | | 0.00 | | 2 | | | 3.85 | | 1 | 0.74 | | | | 6 | | | 1.27 | |
| 15 | 1 | | 3.13 | | 1 | | | 3.13 | | 4 | | | 3.48 | | 3 | | | 4.55 | | 4 | | | 10.00 | | 7 | | | 13.46 | | 3 | 2.22 | | | | 23 | | | 4.87 | |
| 16 | 0 | | 0.00 | | 0 | | | 0.00 | | 0 | | | 0.00 | | 0 | | | 0.00 | | 0 | | | 0.00 | | 1 | | | 1.92 | | 1 | 0.74 | | | | 2 | | | 0.42 | |
| 17 | 0 | | 0.00 | | 0 | | | 0.00 | | 0 | | | 0.00 | | 0 | | | 0.00 | | 0 | | | 0.00 | | 1 | | | 1.92 | | 0 | 0.00 | | | | 1 | | | 0.21 | |
| 18 | 0 | | 0.00 | | 0 | | | 0.00 | | 1 | | | 0.87 | | 0 | | | 0.00 | | 0 | | | 0.00 | | 1 | | | 1.92 | | 0 | 0.00 | | | | 2 | | | 0.42 | |
| 19 | 0 | | 0.00 | | 0 | | | 0.00 | | 0 | | | 0.00 | | 0 | | | 0.00 | | 0 | | | 0.00 | | 1 | | | 1.92 | | 1 | 0.74 | | | | 2 | | | 0.42 | |
| 20 | 0 | | 0.00 | | 0 | | | 0.00 | | 2 | | | 1.74 | | 2 | | | 3.03 | | 4 | | | 10.00 | | 1 | | | 1.92 | | 4 | 2.96 | | | | 13 | | | 2.75 | |
| 21 | 0 | | 0.00 | | 2 | | | 6.25 | | 1 | | | 0.87 | | 0 | | | 0.00 | | 0 | | | 0.00 | | 0 | | | 0.00 | | 0 | 0.00 | | | | 3 | | | 0.64 | |
| 22 | 0 | | 0.00 | | 0 | | | 0.00 | | 0 | | | 0.00 | | 0 | | | 0.00 | | 0 | | | 0.00 | | 0 | | | 0.00 | | 1 | 0.74 | | | | 1 | | | 0.21 | |
| 23 | 0 | | 0.00 | | 0 | | | 0.00 | | 0 | | | 0.00 | | 0 | | | 0.00 | | 0 | | | 0.00 | | 0 | | | 0.00 | | 1 | 0.74 | | | | 1 | | | 0.21 | |
| 24 | 1 | | 3.13 | | 0 | | | 0.00 | | 0 | | | 0.00 | | 0 | | | 0.00 | | 0 | | | 0.00 | | 0 | | | 0.00 | | 0 | 0.00 | | | | 1 | | | 0.21 | |
| 25 | 0 | | 0.00 | | 0 | | | 0.00 | | 1 | | | 0.87 | | 0 | | | 0.00 | | 2 | | | 5.00 | | 2 | | | 3.85 | | 2 | 1.48 | | | | 7 | | | 1.48 | |
| 26 | 0 | | 0.00 | | 0 | | | 0.00 | | 0 | | | 0.00 | | 0 | | | 0.00 | | 1 | | | 2.50 | | 0 | | | 0.00 | | 1 | 0.74 | | | | 2 | | | 0.42 | |
| 28 | 1 | | 3.13 | | 0 | | | 0.00 | | 0 | | | 0.00 | | 0 | | | 0.00 | | 0 | | | 0.00 | | 0 | | | 0.00 | | 1 | 0.74 | | | | 2 | | | 0.42 | |
| 30 | 1 | | 3.13 | | 1 | | | 3.13 | | 1 | | | 0.87 | | 0 | | | 0.00 | | 6 | | | 15.00 | | 0 | | | 0.00 | | 3 | 2.22 | | | | 12 | | | 2.54 | |
| 31 | 1 | | 3.13 | | 0 | | | 0.00 | | 0 | | | 0.00 | | 0 | | | 0.00 | | 0 | | | 0.00 | | 0 | | | 0.00 | | 1 | 0.74 | | | | 2 | | | 0.42 | |
| 32 | 0 | | 0.00 | | 0 | | | 0.00 | | 0 | | | 0.00 | | 0 | | | 0.00 | | 0 | | | 0.00 | | 0 | | | 0.00 | | 1 | 0.74 | | | | 1 | | | 0.21 | |
| 35 | 0 | | 0.00 | | 0 | | | 0.00 | | 1 | | | 0.87 | | 0 | | | 0.00 | | 0 | | | 0.00 | | 0 | | | 0.00 | | 3 | 2.22 | | | | 4 | | | 0.85 | |
| 38 | 0 | | 0.00 | | 0 | | | 0.00 | | 0 | | | 0.00 | | 1 | | | 1.52 | | 0 | | | 0.00 | | 0 | | | 0.00 | | 0 | 0.00 | | | | 1 | | | 0.21 | |
| 40 | 0 | | 0.00 | | 0 | | | 0.00 | | 2 | | | 1.74 | | 1 | | | 1.52 | | 1 | | | 2.50 | | 1 | | | 1.92 | | 2 | 1.48 | | | | 7 | | | 1.48 | |
| 42 | 0 | | 0.00 | | 1 | | | 3.13 | | 0 | | | 0.00 | | 0 | | | 0.00 | | 0 | | | 0.00 | | 0 | | | 0.00 | | 0 | 0.00 | | | | 1 | | | 0.21 | |
| 43 | 0 | | 0.00 | | 1 | | | 3.13 | | 0 | | | 0.00 | | 0 | | | 0.00 | | 0 | | | 0.00 | | 0 | | | 0.00 | | 0 | 0.00 | | | | 1 | | | 0.21 | |
| 45 | 1 | | 3.13 | | 1 | | | 3.13 | | 1 | | | 0.87 | | 0 | | | 0.00 | | 0 | | | 0.00 | | 3 | | | 5.77 | | 0 | 0.00 | | | | 6 | | | 1.27 | |
| 47 | 0 | | 0.00 | | 0 | | | 0.00 | | 0 | | | 0.00 | | 1 | | | 1.52 | | 0 | | | 0.00 | | 0 | | | 0.00 | | 0 | 0.00 | | | | 1 | | | 0.21 | |
| 48 | 0 | | 0.00 | | 0 | | | 0.00 | | 0 | | | 0.00 | | 0 | | | 0.00 | | 0 | | | 0.00 | | 0 | | | 0.00 | | 2 | 1.48 | | | | 2 | | | 0.42 | |
| 50 | 0 | | 0.00 | | 1 | | | 3.13 | | 1 | | | 0.87 | | 0 | | | 0.00 | | 0 | | | 0.00 | | 0 | | | 0.00 | | 2 | 1.48 | | | | 4 | | | 0.85 | |
| 59 | 0 | | 0.00 | | 0 | | | 0.00 | | 0 | | | 0.00 | | 0 | | | 0.00 | | 0 | | | 0.00 | | 0 | | | 0.00 | | 1 | 0.74 | | | | 1 | | | 0.21 | |
| 60 | 0 | | 0.00 | | 0 | | | 0.00 | | 0 | | | 0.00 | | 0 | | | 0.00 | | 0 | | | 0.00 | | 1 | | | 1.92 | | 1 | 0.74 | | | | 2 | | | 0.42 | |
| 65 | 0 | | 0.00 | | 0 | | | 0.00 | | 0 | | | 0.00 | | 0 | | | 0.00 | | 0 | | | 0.00 | | 1 | | | 1.92 | | 0 | 0.00 | | | | 1 | | | 0.21 | |
| N/A | 1 | | 3.13 | | 1 | | | 3.13 | | 0 | | | 0.00 | | 0 | | | 0.00 | | 0 | | | 0.00 | | 2 | | | 3.85 | | 0 | 0.00 | | | | 4 | | | 0.85 | |
| Total | **32** | |  | | **32** | | |  | | **115** | | |  | | **66** | | |  | | **40** | | |  | | **52** | | |  | | **135** |  | | | | **472** | | |  | |
| B: Years as professionals | | **Estonia** | | | | | **Germany** | | | | | **Ireland** | | | | | **Italy** | | | | | **Spain** | | | | | **Switzerland** | | | | | | **UK** | | | | **Total** | | |
|  |  | **n** | | **%** | | **n** | | | **%** | | **n** | | | **%** | | **n** | | | **%** | | **n** | | | **%** | | **n** | | | **%** | | | **n** | | **%** | | **n** | | | **%** |
| 0 | | 14 | | 43.75 | | 29 | | | 87.88 | | 103 | | | 89.57 | | 25 | | | 37.88 | | 22 | | | 55.00 | | 43 | | | 82.69 | | | 116 | | 85.29 | | 352 | | | 74.26 |
| 1 | | 0 | | 0.00 | | 1 | | | 3.03 | | 2 | | | 1.74 | | 5 | | | 7.58 | | 4 | | | 10.00 | | 0 | | | 0.00 | | | 4 | | 2.94 | | 17 | | | 3.59 |
| 2 | | 1 | | 3.13 | | 1 | | | 3.03 | | 2 | | | 1.74 | | 1 | | | 1.52 | | 2 | | | 5.00 | | 1 | | | 1.92 | | | 3 | | 2.21 | | 11 | | | 2.32 |
| 3 | | 1 | | 3.13 | | 0 | | | 0.00 | | 1 | | | 0.87 | | 6 | | | 0.09 | | 4 | | | 10.00 | | 2 | | | 3.85 | | | 5 | | 3.68 | | 19 | | | 4.01 |
| 4 | | 1 | | 3.13 | | 0 | | | 0.00 | | 0 | | | 0.00 | | 2 | | | 3.03 | | 1 | | | 2.50 | | 0 | | | 0.00 | | | 0 | | 0.00 | | 4 | | | 0.84 |
| 5 | | 4 | | 12.50 | | 0 | | | 0.00 | | 3 | | | 2.61 | | 3 | | | 4.55 | | 0 | | | 0.00 | | 1 | | | 1.92 | | | 1 | | 0.74 | | 12 | | | 2.53 |
| 6 | | 1 | | 3.13 | | 0 | | | 0.00 | | 0 | | | 0.00 | | 1 | | | 1.52 | | 0 | | | 0.00 | | 0 | | | 0.00 | | | 0 | | 0.00 | | 2 | | | 0.42 |
| 7 | | 1 | | 3.13 | | 0 | | | 0.00 | | 0 | | | 0.00 | | 4 | | | 6.06 | | 0 | | | 0.00 | | 0 | | | 0.00 | | | 0 | | 0.00 | | 5 | | | 1.05 |
| 8 | | 0 | | 0.00 | | 0 | | | 0.00 | | 1 | | | 0.87 | | 1 | | | 1.52 | | 0 | | | 0.00 | | 1 | | | 1.92 | | | 1 | | 0.74 | | 4 | | | 0.84 |
| 9 | | 0 | | 0.00 | | 0 | | | 0.00 | | 0 | | | 0.00 | | 0 | | | 0.00 | | 1 | | | 2.50 | | 1 | | | 1.92 | | | 0 | | 0.00 | | 2 | | | 0.42 |
| 10 | | 1 | | 3.13 | | 0 | | | 0.00 | | 1 | | | 0.87 | | 5 | | | 7.58 | | 1 | | | 2.50 | | 0 | | | 0.00 | | | 1 | | 0.74 | | 9 | | | 1.90 |
| 11 | | 1 | | 3.13 | | 0 | | | 0.00 | | 0 | | | 0.00 | | 1 | | | 1.52 | | 0 | | | 0.00 | | 0 | | | 0.00 | | | 0 | | 0.00 | | 2 | | | 0.42 |
| 12 | | 0 | | 0.00 | | 0 | | | 0.00 | | 0 | | | 0.00 | | 0 | | | 0.00 | | 1 | | | 2.50 | | 0 | | | 0.00 | | | 0 | | 0.00 | | 1 | | | 0.21 |
| 13 | | 0 | | 0.00 | | 1 | | | 3.03 | | 0 | | | 0.00 | | 0 | | | 0.00 | | 0 | | | 0.00 | | 0 | | | 0.00 | | | 0 | | 0.00 | | 1 | | | 0.21 |
| 15 | | 1 | | 3.13 | | 0 | | | 0.00 | | 1 | | | 0.87 | | 0 | | | 0.00 | | 1 | | | 2.50 | | 0 | | | 0.00 | | | 0 | | 0.00 | | 3 | | | 0.63 |
| 17 | | 0 | | 0.00 | | 0 | | | 0.00 | | 0 | | | 0.00 | | 0 | | | 0.00 | | 1 | | | 2.50 | | 0 | | | 0.00 | | | 0 | | 0.00 | | 1 | | | 0.21 |
| 18 | | 0 | | 0.00 | | 0 | | | 0.00 | | 0 | | | 0.00 | | 1 | | | 1.52 | | 0 | | | 0.00 | | 0 | | | 0.00 | | | 0 | | 0.00 | | 1 | | | 0.21 |
| 21 | | 0 | | 0.00 | | 0 | | | 0.00 | | 0 | | | 0.00 | | 1 | | | 1.52 | | 0 | | | 0.00 | | 0 | | | 0.00 | | | 0 | | 0.00 | | 1 | | | 0.21 |
| 22 | | 0 | | 0.00 | | 0 | | | 0.00 | | 0 | | | 0.00 | | 1 | | | 1.52 | | 0 | | | 0.00 | | 0 | | | 0.00 | | | 0 | | 0.00 | | 1 | | | 0.21 |
| 25 | | 0 | | 0.00 | | 0 | | | 0.00 | | 0 | | | 0.00 | | 1 | | | 1.52 | | 0 | | | 0.00 | | 0 | | | 0.00 | | | 0 | | 0.00 | | 1 | | | 0.21 |
| 30 | | 1 | | 3.13 | | 0 | | | 0.00 | | 1 | | | 0.87 | | 3 | | | 4.55 | | 0 | | | 0.00 | | 0 | | | 0.00 | | | 3 | | 2.21 | | 8 | | | 1.69 |
| 35 | | 1 | | 3.13 | | 0 | | | 0.00 | | 0 | | | 0.00 | | 2 | | | 3.03 | | 0 | | | 0.00 | | 0 | | | 0.00 | | | 0 | | 0.00 | | 3 | | | 0.63 |
| 36 | | 1 | | 3.13 | | 0 | | | 0.00 | | 0 | | | 0.00 | | 0 | | | 0.00 | | 0 | | | 0.00 | | 0 | | | 0.00 | | | 0 | | 0.00 | | 1 | | | 0.21 |
| 37 | | 0 | | 0.00 | | 0 | | | 0.00 | | 0 | | | 0.00 | | 0 | | | 0.00 | | 1 | | | 2.50 | | 0 | | | 0.00 | | | 0 | | 0.00 | | 1 | | | 0.21 |
| 42 | | 0 | | 0.00 | | 0 | | | 0.00 | | 0 | | | 0.00 | | 1 | | | 1.52 | | 0 | | | 0.00 | | 0 | | | 0.00 | | | 0 | | 0.00 | | 1 | | | 0.21 |
| 45 | | 1 | | 3.13 | | 0 | | | 0.00 | | 0 | | | 0.00 | | 1 | | | 1.52 | | 1 | | | 2.50 | | 0 | | | 0.00 | | | 0 | | 0.00 | | 3 | | | 0.63 |
| 50 | | 0 | | 0.00 | | 0 | | | 0.00 | | 0 | | | 0.00 | | 0 | | | 0.00 | | 0 | | | 0.00 | | 0 | | | 0.00 | | | 1 | | 0.74 | | 1 | | | 0.21 |
| N/A | | 2 | | 6.25 | | 1 | | | 3.03 | | 0 | | | 0.00 | | 1 | | | 1.52 | | 0 | | | 0.00 | | 3 | | | 5.77 | | | 1 | | 0.74 | | 8 | | | 1.69 |
| Total | | **32** | |  | | **33** | | |  | | **115** | | |  | | **66** | | |  | | **40** | | |  | | **52** | | |  | | | **136** | |  | | **474** | | |  |

| Table D1. Reasons to practice beekeeping. To this multiple choice question, nearly 80% of beekeepers selected “personal hobby” among the reasons to practice beekeeping. Out of 14% beekeepers selecting “other”, sixty-five (13.71% of all respondents and 89% of those selecting “other”) listed additional reasons, which are reported below. Reasons suggested more frequently are highlighted in bold. | | |
| --- | --- | --- |
| Reasons to practice beekeeping (N = 474) | **N** | **%** |
| Awareness of threats to pollinators | 190 | 36.61 |
| Environmental concerns | 198 | 38.15 |
| Personal hobby | **400** | **77.07** |
| Providing paid pollination services to growers | 22 | 4.24 |
| Selling honey, beeswax, pollen, other products | 233 | 44.89 |
| Other | 73 | 14.07 |
| Other reasons to practice beekeeping (n = 65) | **N** | **%** |
| Sustainability | 1 | 1.54 |
| Enjoyment | 3 | 4.62 |
| Bee health | 4 | 6.15 |
| Teaching/helping other beekeepers | 4 | 6.15 |
| Make own products for personal use | 5 | 7.69 |
| Self-learning | 10 | 15.38 |
| Own farm pollination | 2 | 3.08 |
| Make own products as gift | 4 | 3.08 |
| Job | 6 | 9.23 |
| Conservation | 3 | 4.62 |
| Own garden pollination | 3 | 4.62 |
| Crop pollination | 3 | 4.62 |
| Fascination for bees/nature | **18** | **27.69** |
| Queen rearing | 1 | 1.54 |
| Inheritance | 6 | 9.23 |
| Selling bees | 1 | 1.54 |
| Environmental concerns | 2 | 3.08 |

| Table D2. Reasons to practice beekeeping per country. People practicing beekeeping because it is a personal hobby overcome 90% in Germany and Switzerland, and 80% in Spain and the UK. The most selected reason to practice beekeeping in Italy is “selling honey, beeswax, pollen, other products”, while in Estonia is equally “personal hobby” and “selling honey, beeswax, pollen, other products”. Such results are in line with Table B above, showing that the majority of professional beekeepers participating in the survey are based in Italy (60.61%) and Estonia (50%). Reasons suggested more frequently in each country are highlighted in bold. | | | | | | | |
| --- | --- | --- | --- | --- | --- | --- | --- |
| Reasons to practice beekeeping (N = 474) | **Estonia** | **Germany** | **Ireland** | **Italy** | **Spain** | **Switzerland** | **UK** |
| Awareness of threats to pollinators | 18.75% | 51.52% | 35.42% | 42.86% | 22.50% | 32.69% | 40.54% |
| Environmental concerns | 12.50% | 30.30% | 45.83% | 27.14% | 40.00% | 40.38% | 41.89% |
| Personal hobby | **71.88%** | **93.94%** | **71.53%** | 47.14% | **82.50%** | **90.38%** | **87.84%** |
| Providing paid pollination services to growers | 6.25% | 12.12% | 2.78% | 4.29% | 0.00% | 9.62% | 2.70% |
| Selling honey, beeswax, pollen, other products | **71.88%** | 57.58% | 27.78% | **67.14%** | 47.50% | 57.69% | 37.16% |
| Other | 21.88% | 18.18% | 8.33% | 12.86% | 12.50% | 19.23% | 16.22% |

**Beekeepers’ knowledge exchange**

| Table E1. Frequency of communication with growers. The highest percentages per country are highlighted in bold. | | | | | | | | |
| --- | --- | --- | --- | --- | --- | --- | --- | --- |
| Communication with growers | **Country** | | | | | | | |
|  | **Estonia** | **Germany** | **Ireland** | **Italy** | **Spain** | **Switzerland** | **UK** | **Total** |
| Frequent communication (more than twice a year) | 21.88% | 27.27% | 17.39% | **40.91%** | **47.50%** | **57.69%** | 12.50% | 27.22% |
| I am a grower myself and manage my own hives on my lands | 15.63% | 0.00% | 7.83% | 19.70% | 20.00% | 3.85% | 11.76% | 11.18% |
| I do not communicate with growers | 21.88% | **39.39%** | **62.61%** | 18.18% | 22.50% | 3.85% | **67.65%** | **43.67%** |
| Infrequent communication (once or twice a year) | **40.63%** | 33.33% | 12.17% | 21.21% | 10.00% | 32.69% | 8.09% | 17.72% |
| Only when taking payments for professional pollination services | 0.00% | 0.00% | 0.00% | 0.00% | 0.00% | 1.92% | 0.00% | 0.21% |

| Table E2. Frequency of communication with growers in hobbyist and professional beekeepers. More than 50% hobbyist beekeepers do not communicate with growers at all, while nearly 43% professional beekeepers communicate with growers more than twice a year. The highest percentages per beekeeper type are highlighted in bold. | | |
| --- | --- | --- |
|  | **Hobbyists** | **Professionals** |
| Frequent communication (more than twice a year) | 22.16% | **42.98%** |
| I am a grower myself and manage my own hives on my lands | 8.81% | 19.30% |
| I do not communicate with growers | **51.42%** | 18.42% |
| Infrequent communication (once or twice a year) | 17.33% | 19.30% |
| Only when taking payments for professional pollination services | 0.28% | 0.00% |

| Table F. Beekeepers’ sources of information on beehive health. The highest percentages per source per country are highlighted in bold. | | | | | | |
| --- | --- | --- | --- | --- | --- | --- |
| Country | **Source of information** | **Importance of sources of information** | | | | |
|  |  | **Extremely important** | **Very important** | **Moderately important** | **Slightly important** | **Not at all important** |
| Estonia | Beekeeping associations | 15.63% | **43.75%** | 31.25% | 9.38% | 0.00% |
|  | Other beekeepers | 18.75% | **37.50%** | **37.50%** | 6.25% | 0.00% |
|  | Training in person | 6.25% | **34.38%** | 31.25% | 21.88% | 6.25% |
|  | National bee health agencies | 3.13% | 21.88% | **31.25%** | 12.50% | 31.25% |
|  | Journals | **28.13%** | 18.75% | 25.00% | 21.88% | 6.25% |
|  | Social media | 0.00% | 15.63% | 28.13% | **37.50%** | 18.75% |
|  | Online training | 3.13% | 15.63% | 28.13% | **37.50%** | 15.63% |
|  | Newspapers | 6.25% | 28.13% | **37.50%** | 21.88% | 6.25% |
|  | TV/Radio | 3.13% | 6.25% | 31.25% | **34.38%** | 25.00% |
|  | NGOs | 3.13% | 3.13% | **31.25%** | 25.00% | 37.50% |
| Germany | Beekeeping associations | 27.27% | **39.39%** | 18.18% | 12.12% | 3.03% |
|  | Other beekeepers | **39.39%** | **39.39%** | 12.12% | 3.03% | 6.06% |
|  | Training in person | 15.15% | **51.52%** | 12.12% | 9.09% | 12.12% |
|  | National bee health agencies | 12.12% | 21.21% | 15.15% | 18.18% | **33.33%** |
|  | Journals | 15.15% | **48.48%** | 9.09% | 15.15% | 12.12% |
|  | Social media | 3.03% | 9.09% | 15.15% | 24.24% | **48.48%** |
|  | Online training | 6.06% | 9.09% | 18.18% | 24.24% | **42.42%** |
|  | Newspapers | 6.06% | 24.24% | 27.27% | **30.30%** | 12.12% |
|  | TV/Radio | 0.00% | 6.06% | 15.15% | **39.39%** | **39.39%** |
|  | NGOs | 3.03% | 9.09% | 9.09% | 18.18% | **60.61%** |
| Ireland | Beekeeping associations | **54.78%** | 34.78% | 6.09% | 3.48% | 0.87% |
|  | Other beekeepers | 41.74% | **42.61%** | 11.30% | 2.61% | 1.74% |
|  | Training in person | 33.91% | **38.26%** | 14.78% | 6.09% | 6.96% |
|  | National bee health agencies | 31.30% | **35.65%** | 13.91% | 10.43% | 8.70% |
|  | Journals | 19.13% | **27.83%** | 23.48% | 16.52% | 13.04% |
|  | Social media | 13.91% | 20.87% | **21.74%** | **21.74%** | **21.74%** |
|  | Online training | 10.43% | 22.61% | 21.74% | 17.39% | **27.83%** |
|  | Newspapers | 7.83% | 23.48% | **26.96%** | 20.00% | 21.74% |
|  | TV/Radio | 8.70% | 6.96% | 21.74% | 30.43% | **32.17%** |
|  | NGOs | 6.96% | 12.17% | 20.00% | 26.96% | **33.91%** |
| Italy | Beekeeping associations | **45.45%** | 33.33% | 15.15% | 4.55% | 1.52% |
|  | Other beekeepers | **40.91%** | 33.33% | 18.18% | 6.06% | 1.52% |
|  | Training in person | **42.42%** | 39.39% | 13.64% | 4.55% | 0.00% |
|  | National bee health agencies | 21.21% | **31.82%** | 25.76% | 12.12% | 9.09% |
|  | Journals | 30.30% | **50.00%** | 15.15% | 4.55% | 0.00% |
|  | Social media | 7.58% | 21.21% | **40.91%** | 21.21% | 9.09% |
|  | Online training | 22.73% | **33.33%** | 28.79% | 10.61% | 4.55% |
|  | Newspapers | 13.64% | **37.88%** | 28.79% | 13.64% | 6.06% |
|  | TV/Radio | 6.06% | 16.67% | 18.18% | **36.36%** | 22.73% |
|  | NGOs | 12.12% | 9.09% | **28.79%** | **28.79%** | 21.21% |
| Spain | Beekeeping associations | 35.00% | **47.50%** | 12.50% | 5.00% | 0.00% |
|  | Other beekeepers | 27.50% | **42.50%** | 15.00% | 12.50% | 2.50% |
|  | Training in person | 32.50% | **47.50%** | 15.00% | 5.00% | 0.00% |
|  | National bee health agencies | 10.00% | **37.50%** | 32.50% | 12.50% | 7.50% |
|  | Journals | 17.50% | **37.50%** | 20.00% | 17.50% | 7.50% |
|  | Social media | 7.50% | 25.00% | **40.00%** | 17.50% | 10.00% |
|  | Online training | 17.50% | **37.50%** | 35.00% | 10.00% | 0.00% |
|  | Newspapers | 0.00% | 25.00% | 27.50% | **35.00%** | 12.50% |
|  | TV/Radio | 2.50% | 12.50% | 30.00% | **32.50%** | 22.50% |
|  | NGOs | 2.50% | 5.00% | 12.50% | **37.50%** | 42.50% |
| Switzerland | Beekeeping associations | **50.00%** | 38.46% | 7.69% | 3.85% | 0.00% |
|  | Other beekeepers | 28.85% | **53.85%** | 5.77% | 9.62% | 1.92% |
|  | Training in person | **63.46%** | 32.69% | 1.92% | 0.00% | 1.92% |
|  | National bee health agencies | **46.15%** | 42.31% | 9.62% | 0.00% | 1.92% |
|  | Journals | **28.13%** | 18.75% | 25.00% | 21.88% | 6.25% |
|  | Social media | 0.00% | 23.08% | 23.08% | **30.77%** | 23.08% |
|  | Online training | 11.54% | **30.77%** | **30.77%** | 15.38% | 11.54% |
|  | Newspapers | 17.31% | **30.77%** | 26.92% | 15.38% | 9.62% |
|  | TV/Radio | 1.92% | 15.38% | **30.77%** | 28.85% | 23.08% |
|  | NGOs | 3.85% | 19.23% | 28.85% | 11.54% | **36.54%** |
| UK | Beekeeping associations | **47.06%** | 30.88% | 13.97% | 5.15% | 2.94% |
|  | Other beekeepers | **42.65%** | 33.82% | 15.44% | 3.68% | 4.41% |
|  | Training in person | 28.68% | **38.24%** | 16.18% | 8.09% | 8.82% |
|  | National bee health agencies | 25.74% | **39.71%** | 22.06% | 7.35% | 5.15% |
|  | Journals | 18.38% | 19.85% | **25.00%** | 17.50% | 17.65% |
|  | Social media | 9.56% | 8.09% | 22.79% | 20.59% | **38.97%** |
|  | Online training | 8.82% | 17.65% | **30.15%** | 20.59% | 22.79% |
|  | Newspapers | 5.15% | 11.76% | 19.85% | 17.65% | **45.59%** |
|  | TV/Radio | 7.35% | 0.74% | 17.65% | 25.00% | **49.26%** |
|  | NGOs | 10.29% | 13.24% | **30.88%** | 18.38% | 27.21% |

**Bee decline**

| Table G. Respondents’ reasons of bee decline. The highest percentages per reason per country are highlighted in bold. | | | | | | | |
| --- | --- | --- | --- | --- | --- | --- | --- |
| Country | **Reasons for bee decline** |  | | Agreement | | |  |
|  |  | **Strongly agree** | **Agree** | | **Neutral** | **Disagree** | **Strongly disagree** |
| Estonia | Loss of natural habitats | 25.00% | **40.63%** | | 21.88% | 9.38% | 3.13% |
|  | Competition wild/managed | 3.13% | 15.63% | | 25.00% | **37.50%** | 18.75% |
|  | Diseases | 40.63% | **53.13%** | | 3.13% | 3.13% | 0.00% |
|  | Parasites | **56.25%** | 34.38% | | 6.25% | 3.13% | 0.00% |
|  | Predators | 12.50% | 21.88% | | **34.38%** | 31.25% | 0.00% |
|  | Climate change | 6.25% | 34.38% | | **40.63%** | 12.50% | 6.25% |
|  | Genetics | 3.13% | 34.38% | | **46.88%** | 15.63% | 0.00% |
|  | Non-optimal beekeeping | 31.25% | **50.00%** | | 9.38% | 9.38% | 0.00% |
|  | Agrochemicals | 34.38% | **50.00%** | | 12.50% | 3.13% | 0.00% |
| Germany | Loss of natural habitats | **48.48%** | 42.42% | | 3.03% | 3.03% | 3.03% |
|  | Competition wild/managed | 3.03% | 15.15% | | 30.30% | **42.42%** | 9.09% |
|  | Diseases | 18.18% | **33.33%** | | 30.30% | 15.15% | 3.03% |
|  | Parasites | 30.30% | 30.30% | | **33.33%** | 3.03% | 3.03% |
|  | Predators | 0.00% | 3.03% | | 21.21% | **51.52%** | 24.24% |
|  | Climate change | 12.12% | 21.21% | | **33.33%** | 27.27% | 6.06% |
|  | Genetics | 0.00% | 15.15% | | **39.39%** | 27.27% | 18.18% |
|  | Non-optimal beekeeping | 6.06% | 36.36% | | **30.30%** | 18.18% | 9.09% |
|  | Agrochemicals | 40.63% | **50.00%** | | 6.25% | 3.13% | 0.00% |
| Ireland | Loss of natural habitats | **66.09%** | 28.79% | | 3.48% | 0.87% | 0.87 |
|  | Competition wild/managed | 3.48% | 11.30% | | **52.17%** | 22.61% | 10.43% |
|  | Diseases | 22.61% | **53.04%** | | 22.61% | 0.87% | 0.87% |
|  | Parasites | 32.17% | **47.83%** | | 17.39% | 2.61% | 0.00% |
|  | Predators | 1.74% | 13.04% | | **44.35%** | 33.91% | 6.96% |
|  | Climate change | 15.65% | **43.48%** | | 33.04% | 7.83% | 0.00% |
|  | Genetics | 4.35% | 20.87% | | **56.52%** | 18.26% | 0.00% |
|  | Non-optimal beekeeping | 9.57% | 30.43% | | **46.96%** | 11.30% | 1.74% |
|  | Agrochemicals | **65.22%** | 30.43% | | 2.61% | 1.74% | 0.00% |
| Italy | Loss of natural habitats | **62.12%** | 36.36% | | 0.00% | 1.52% | 0.00% |
|  | Competition wild/managed | 12.12% | 10.61% | | **36.36%** | 30.30% | 10.61% |
|  | Diseases | **40.91%** | 37.88% | | 16.67% | 4.55% | 0.00% |
|  | Parasites | **50.00%** | 39.39% | | 7.58% | 3.03% | 0.00% |
|  | Predators | 13.64% | **33.33%** | | 24.24% | 21.21% | 7.58% |
|  | Climate change | **63.64%** | 25.76% | | 7.58% | 3.03% | 0.00% |
|  | Genetics | 4.55% | 16.67% | | **43.94%** | 30.30% | 4.55% |
|  | Non-optimal beekeeping | 15.15% | **39.39%** | | 25.76% | 12.12% | 7.58% |
|  | Agrochemicals | **75.76%** | 18.18% | | 6.06% | 0.00% | 0.00% |
| Spain | Loss of natural habitats | 40.00% | **45.00**% | | 7.50% | 5.00% | 2.5% |
|  | Competition wild/managed | 7.50% | 17.50% | | 15.00% | **35.00%** | 25.00% |
|  | Diseases | **57.50%** | 27.50% | | 12.50% | 2.50% | 0.00% |
|  | Parasites | **57.50%** | 30.00% | | 12.50% | 0.00% | 0.00% |
|  | Predators | 12.50% | **30.00%** | | 27.50% | 25.00% | 5.00% |
|  | Climate change | **47.50%** | 35.00% | | 10.00% | 5.00% | 2.50% |
|  | Genetics | 10.00% | 22.50% | | **30.00%** | **30.30%** | 7.50% |
|  | Non-optimal beekeeping | 22.50% | **35.00%** | | 22.50% | 12.50% | 7.50% |
|  | Agrochemicals | **57.50%** | 32.50% | | 7.50% | 2.50% | 0.00% |
| Switzerland | Loss of natural habitats | **61.54%** | 28.85% | | 3.85% | 3.85% | 1.92% |
|  | Competition wild/managed | 3.85% | 9.62% | | 30.77% | **40.38%** | 15.38% |
|  | Diseases | 17.31% | **42.31%** | | 19.23% | 19.23% | 1.92% |
|  | Parasites | **36.54%** | 28.85 | | 15.38% | 17.31% | 4.01% |
|  | Predators | 1.92% | 3.85% | | 25.00% | **42.31%** | 26.92% |
|  | Climate change | 9.62% | **30.77%** | | 21.15% | 26.92% | 11.54% |
|  | Genetics | 7.69% | 21.15% | | **30.77%** | 28.85% | 11.54% |
|  | Non-optimal beekeeping | 23.08% | **42.31%** | | 26.92% | 7.69% | 0.00% |
|  | Agrochemicals | 32.00% | **48.00%** | | 14.00% | 6.00% | 0.00% |
| UK | Loss of natural habitats | **67.65%** | 22.06% | | 7.35% | 1.47% | 1.47% |
|  | Competition wild/managed | 5.88% | 21.32% | | **40.44%** | 23.53% | 8.82% |
|  | Diseases | 26.47% | **51.47%** | | 18.38% | 2.94% | 0.74% |
|  | Parasites | 38.24% | **44.85%** | | 13.97% | 2.21% | 0.74% |
|  | Predators | 4.41% | 30.88% | | **35.29%** | 19.85% | 9.56% |
|  | Climate change | 22.06% | **40.44%** | | 22.79% | 11.76% | 2.94% |
|  | Genetics | 8.09% | 25.74% | | **49.26%** | 14.71% | 2.21% |
|  | Non-optimal beekeeping | 17.65% | **38.24%** | | 35.29% | 6.62% | 2.21% |
|  | Agrochemicals | **53.33%** | 35.56% | | 8.89% | 2.22% | 0.00% |

| Table H. Respondents’ reasons to reduce bee decline. The highest percentages per reason per country are highlighted in bold. | | | | | | | | |
| --- | --- | --- | --- | --- | --- | --- | --- | --- |
| Country | **Reasons to reduce bee decline** | | **Agreement** | | | | | |
|  |  | | **Strongly agree** | **Agree** | | **Neutral** | **Disagree** | **Strongly disagree** |
| Estonia | Collab with growers | | 41.94% | **48.39%** | | 9.68% | 0.00% | 0.00% |
|  | Hive position | | 40.63% | **59.38%** | | 0.00% | 0.00% | 0.00% |
|  | Natural habitats/flower areas | | **53.13%** | 40.63% | | 6.25% | 0.00% | 0.00% |
|  | Monitor diseases | | **59.38%** | 37.50% | | 3.13% | 0.00% | 0.00% |
|  | Monitor parasites | | **68.75%** | 25.00% | | 6.25% | 0.00% | 0.00% |
|  | Monitor nutrition | | 28.13% | **46.88%** | | 21.88% | 3.13% | 0.00% |
|  | Monitor agrochemicals | | 37.50% | **46.88%** | | 15.63% | 0.00% | 0.00% |
|  | Optimal beekeeping | | 40.63% | **53.13%** | | 6.25% | 0.00% | 0.00% |
| Germany | Collab with growers | | **54.55%** | 39.39% | | 3.03% | 0.00% | 3.03% |
|  | Hive position | | 32.26% | **45.16%** | | 16.13% | 6.45% | 0.00% |
|  | Natural habitats/flower areas | | **65.63%** | 31.25% | | 3.13% | 0.00% | 0.00% |
|  | Monitor diseases | | 37.50% | **40.63%** | | 21.88% | 0.00% | 0.00% |
|  | Monitor parasites | | **46.88%** | 28.13% | | 25.00% | 0.00% | 0.00% |
|  | Monitor nutrition | | 15.15% | **54.55%** | | 27.27% | 0.00% | 3.03% |
|  | Monitor agrochemicals | | **53.13%** | 34.38% | | 12.50% | 53.13% | 0.00% |
|  | Optimal beekeeping | | 35.48% | **51.61%** | | 12.90% | 0.00% | 0.00% |
| Ireland | Collab with growers | | **46.09%** | **46.09%** | | 7.83% | 0.00% | 0.00% |
|  | Hive position | | 26.96% | **55.65%** | | 14.78% | 2.61% | 0.00% |
|  | Natural habitats/flower areas | | **62.61%** | 33.91% | | 1.74% | 1.74% | 0.00% |
|  | Monitor diseases | | 43.48% | **53.04%** | | 3.48% | 0.00% | 0.00% |
|  | Monitor parasites | | 44.35% | **51.30%** | | 4.35% | 0.00% | 0.00% |
|  | Monitor nutrition | | 39.13% | **43.48%** | | 15.65% | 0.87% | 0.87% |
|  | Monitor agrochemicals | | **68.70%** | 26.09% | | 4.35% | 0.87% | 0.00% |
|  | Optimal beekeeping | | **42.61%** | 41.74% | | 15.65% | 0.00% | 0.00% |
| Italy | Collab with growers | | **60.94%** | 31.25% | | 7.81% | 0.00% | 0.00% |
|  | Hive position | | 39.06% | **46.88%** | | 14.06% | 0.00% | 0.00% |
|  | Natural habitats/flower areas | | **74.24%** | 24.24% | | 0.00% | 1.52% | 0.00% |
|  | Monitor diseases | | **45.31%** | 42.19% | | 12.50% | 0.00% | 0.00% |
|  | Monitor parasites | | **48.44%** | 45.31% | | 6.25% | 0.00% | 0.00% |
|  | Monitor nutrition | | 34.85% | **40.91%** | | 19.70% | 4.55% | 0.00% |
|  | Monitor agrochemicals | | **66.67%** | 30.30% | | 3.03% | 0.00% | 0.00% |
|  | Optimal beekeeping | | **51.52%** | 33.33% | | 15.15% | 0.00% | 0.00% |
| Spain | Collab with growers | | 45.00% | **52.50%** | | 2.50% | 0.00% | 0.00% |
|  | Hive position | | 35.00% | **42.50%** | | 17.50% | 5.00% | 0.00% |
|  | Natural habitats/flower areas | | 35.00% | **45.00%** | | 20.00% | 0.00% | 0.00% |
|  | Monitor diseases | | **60.00%** | 32.50% | | 7.50% | 0.00% | 0.00% |
|  | Monitor parasites | | **57.50%** | 32.50% | | 10.00% | 0.00% | 0.00% |
|  | Monitor nutrition | | **37.50%** | **37.50%** | | 17.50% | 7.50% | 0.00% |
|  | Monitor agrochemicals | | **55.00%** | 37.50% | | 7.50% | 0.00% | 0.00% |
|  | Optimal beekeeping | | **56.41%** | 33.33% | | 10.26% | 0.00% | 0.00% |
| Switzerland | Collab with growers | | **50.00%** | 48.08% | | 1.92% | 0.00% | 0.00% |
|  | Hive position | | **48.08%** | 36.54% | | 7.69% | 7.69% | 0.00% |
|  | Natural habitats/flower areas | | **63.46%** | 30.77% | | 5.77% | 0.00% | 0.00% |
|  | Monitor diseases | | **48.08%** | 40.38% | | 11.54% | 0.00% | 0.00% |
|  | Monitor parasites | | **46.15%** | 42.31% | | 11.54% | 0.00% | 0.00% |
|  | Monitor nutrition | | **42.31%** | 34.62% | | 19.23% | 3.85% | 0.00% |
|  | Monitor agrochemicals | | **50.00%** | 36.54% | | 9.62% | 3.85% | 0.00% |
|  | Optimal beekeeping | | **52.94%** | 45.10% | | 1.96% | 0.00% | 0.00% |
| UK | Collab with growers | | 29.85% | **46.27%** | | 22.39% | 0.00% | 1.49% |
|  | Hive position | | 27.21% | **51.47%** | | 19.12% | 2.21% | 0.00% |
|  | Natural habitats/flower areas | | **71.32%** | 24.26% | | 4.41% | 0.00% | 0.00% |
|  | Monitor diseases | | **54.07%** | 41.48% | | 4.44% | 0.00% | 0.00% |
|  | Monitor parasites | | **53.68%** | 41.18% | | 5.15% | 0.00% | 0.00% |
|  | Monitor nutrition | | **41.91%** | 40.44% | | 17.65% | 0.00% | 0.00% |
|  | Monitor agrochemicals | | **57.04%** | 33.33% | | 8.89% | 0.74% | 0.00% |
|  | Optimal beekeeping | | **54.07%** | 31.11% | | 14.81% | 0.00% | 0.00% |
| Table I. Respondents’ reasons to protect bee health. The highest percentages per reason per country are highlighted in bold. | | | | | | | | |
| Country | **Reasons to protect bee health** | **Agreement** | | | | | | |
|  |  | **Strongly agree** | | | **Agree** | **Neutral** | **Disagree** | **Strongly disagree** |
| Estonia | Economic reasons | 28.13% | | | **43.75%** | 25.00% | 3.13% | 0.00% |
|  | Legal reasons | 18.75% | | | **40.63%** | 31.25% | 9.38% | 0.00% |
|  | Public perception | 15.63% | | | 21.88% | **43.75%** | 12.50% | 6.25% |
|  | Pollinators conservation | **65.63%** | | | 28.13% | 6.25% | 0.00% | 0.00% |
|  | Consumer safety | **43.75%** | | | 40.63% | 15.63% | 0.00% | 0.00% |
|  | Food security | 37.50% | | | **56.25%** | 6.25% | 0.00% | 0.00% |
|  | Crop varieties | 34.38% | | | **43.75%** | 18.75% | 3.13% | 0.00% |
| Germany | Economic reasons | 12.12% | | | **33.33%** | **33.33%** | 15.15% | 6.06% |
|  | Legal reasons | 12.12% | | | 30.30% | **36.36%** | 12.12% | 9.09% |
|  | Public perception | 18.18% | | | 30.30% | **36.36%** | 6.06% | 9.09% |
|  | Pollinators conservation | **59.38%** | | | 37.50% | 3.13% | 0.00% | 0.00% |
|  | Consumer safety | 9.09% | | | 30.30% | **33.33%** | 18.18% | 9.09% |
|  | Food security | 24.24% | | | **33.33%** | 27.27% | 12.12% | 3.03% |
|  | Crop varieties | **51.52%** | | | 36.36% | 9.09% | 3.03% | 0.00% |
| Ireland | Economic reasons | 25.22% | | | **40.87%** | 26.09% | 6.09% | 1.74% |
|  | Legal reasons | 13.91% | | | 31.30% | **36.52%** | 13.91% | 4.35% |
|  | Public perception | 18.26% | | | **33.04%** | 32.17% | 13.91% | 2.61% |
|  | Pollinators conservation | **77.39%** | | | 20.00% | 1.74% | 0.87% | 0.00% |
|  | Consumer safety | 30.43% | | | **44.35%** | 16.52% | 7.83% | 0.87% |
|  | Food security | **56.52%** | | | 33.04% | 7.83% | 1.74% | 0.87% |
|  | Crop varieties | 42.61% | | | **43.48%** | 12.17% | 1.74% | 0.00% |
| Italy | Economic reasons | 25.76% | | | **43.94%** | 25.76% | 3.03% | 1.52% |
|  | Legal reasons | 18.18% | | | 31.82% | **42.42%** | 6.06% | 1.52% |
|  | Public perception | 21.21% | | | 30.30% | **31.82%** | 9.09% | 7.58% |
|  | Pollinators conservation | **81.82%** | | | 13.64% | 3.03% | 1.52% | 0.00% |
|  | Consumer safety | **39.39%** | | | 37.88% | 15.15% | 6.06% | 1.52% |
|  | Food security | **57.58%** | | | 28.79% | 9.09% | 4.55% | 0.00% |
|  | Crop varieties | 39.39% | | | **43.94%** | 13.64% | 3.03% | 0.00% |
| Spain | Economic reasons | 27.50% | | | **35.00%** | 22.50% | 15.00% | 0.00% |
|  | Legal reasons | 10.00% | | | 32.50% | **40.00%** | 12.50% | 5.00% |
|  | Public perception | 10.00% | | | **37.50%** | 35.00% | 15.00% | 2.50% |
|  | Pollinators conservation | **60.00%** | | | 35.00% | 5.00% | 0.00% | 0.00% |
|  | Consumer safety | **40.00%** | | | **40.00%** | 17.50% | 2.50% | 0.00% |
|  | Food security | **42.50%** | | | 40.00% | 15.00% | 2.50% | 0.00% |
|  | Crop varieties | 30.00% | | | **50.00%** | 17.50% | 2.50% | 0.00% |
| Switzerland | Economic reasons | 9.62% | | | **30.77%** | 26.92% | 25.00% | 7.69% |
|  | Legal reasons | 13.46% | | | **48.08%** | 26.92% | 11.54% | 0.00% |
|  | Public perception | 21.15% | | | **48.08%** | 23.08% | 7.69% | 0.00% |
|  | Pollinators conservation | **57.69%** | | | 36.54% | 5.77% | 0.00% | 0.00% |
|  | Consumer safety | 17.31% | | | 28.85% | **44.23%** | 7.69% | 1.92% |
|  | Food security | 23.08% | | | **38.46%** | 30.77% | 7.69% | 0.00% |
|  | Crop varieties | 34.62% | | | **42.31%** | 19.23% | 3.85% | 0.00% |
| UK | Economic reasons | **38.24%** | | | 33.09% | 20.59% | 6.62% | 1.47% |
|  | Legal reasons | 12.50% | | | 28.68% | **47.06%** | 9.56% | 2.21% |
|  | Public perception | 16.18% | | | 34.56% | **38.97%** | 9.56% | 0.74% |
|  | Pollinators conservation | **78.68%** | | | 16.91% | 4.41% | 0.00% | 0.00% |
|  | Consumer safety | 24.26% | | | **36.03%** | 32.35% | 5.88% | 1.47% |
|  | Food security | **52.21%** | | | 33.09% | 11.03% | 2.21% | 1.47% |
|  | Crop varieties | **40.44%** | | | 39.71% | 16.18% | 2.21% | 1.47% |

**Bee health**

| Table J. Frequency of health checks performed by respondents. Frequency varied between countries, with UK and Italy generally checking for pressures more regularly than others. It is worth considering that the frequency of performing health checks may also depend on national policies; for example, despite not being listed as notifiable diseases under EU legislation (EU, 2016), both Italy and the UK consider EFB as notifiable (D.P.R., 2006; Statutory Instruments, 2006), with Italy also adding Nosemosis caused by Nosema Apis (D.P.R., 2006). Thus, the presence of more notifiable diseases in national regulations may drive beekeepers to perform more regular health checks. The highest percentages for each check in each country are highlighted in bold. | | | | | |
| --- | --- | --- | --- | --- | --- |
| Country | **Frequency** | **Checks** | | | |
|  |  | **Diseases** | **Parasites** | **Nutrition** | **Chemicals** |
| Estonia | Weekly | **31.25%** | 25.00% | 28.13% | 18.75% |
|  | Fortnightly | 9.38% | 12.50% | 15.63% | 6.25% |
|  | Monthly | 18.75% | 18.75% | 15.63% | 9.38% |
|  | More than once a year | 21.88% | **34.38%** | **31.25%** | 15.63% |
|  | Yearly | 3.13% | 3.13% | 3.13% | 6.25% |
|  | Only with a reasonable suspicion | 15.63% | 6.25% | 6.25% | **43.75%** |
|  | Never | 0.00% | 0.00% | 0.00% | 0.00% |
| Germany | Weekly | 12.12% | 6.06% | 9.09% | 0.00% |
|  | Fortnightly | 15.15% | 15.15% | 18.18% | 3.03% |
|  | Monthly | 9.09% | 9.09% | 15.15% | 3.03% |
|  | More than once a year | **27.27%** | **45.45%** | **33.33%** | 6.06% |
|  | Yearly | 12.12% | 12.12% | 6.06% | 9.09% |
|  | Only with a reasonable suspicion | 21.21% | 9.09% | 9.09% | **48.48%** |
|  | Never | 3.03% | 3.03% | 9.09% | 30.30% |
| Ireland | Weekly | 20.87% | 14.78% | 32.17% | 10.43% |
|  | Fortnightly | **32.17%** | 26.09% | **34.78%** | 8.70% |
|  | Monthly | 19.13% | 20.00% | 17.39% | 3.48% |
|  | More than once a year | 20.87% | **30.43%** | 4.35% | 2.61% |
|  | Yearly | 0.87% | 1.74% | 0.87% | 2.61% |
|  | Only with a reasonable suspicion | 6.09% | 6.09% | 5.22% | 28.70% |
|  | Never | 0.00% | 0.87% | 5.22% | **43.48%** |
| Italy | Weekly | **37.88%** | **27.27%** | 24.24% | 21.21% |
|  | Fortnightly | 27.27% | 25.76% | **31.82%** | 15.15% |
|  | Monthly | 21.21% | 19.70% | 6.06% | 12.12% |
|  | More than once a year | 9.09% | 22.73% | 18.18% | 10.61% |
|  | Yearly | 0.00% | 0.00% | 1.52% | 7.58% |
|  | Only with a reasonable suspicion | 4.55% | 4.55% | 15.15% | **24.24%** |
|  | Never | 0.00% | 0.00% | 3.03% | 9.09% |
| Spain | Weekly | 7.50% | 5.00% | 10.00% | 10.00% |
|  | Fortnightly | 20.00% | 12.50% | 17.50% | 5.00% |
|  | Monthly | **30.00%** | **40.00%** | 22.50% | 7.50% |
|  | More than once a year | 25.00% | 20.00% | **27.50%** | 10.00% |
|  | Yearly | 12.50% | 17.50% | 5.00% | 2.50% |
|  | Only with a reasonable suspicion | 5.00% | 2.50% | 10.00% | 20.00% |
|  | Never | 0.00% | 2.50% | 7.50% | **45.00%** |
| Switzerland | Weekly | 19.23% | 19.23% | 19.23% | 5.77% |
|  | Fortnightly | **34.62%** | 25.00% | **30.77%** | 9.62% |
|  | Monthly | 25.00% | **32.69%** | 25.00% | 1.92% |
|  | More than once a year | 15.38% | 13.46% | 21.15% | 13.46% |
|  | Yearly | 0.00% | 0.00% | 0.00% | 3.85% |
|  | Only with a reasonable suspicion | 5.77% | 9.62% | 3.85% | **46.15%** |
|  | Never | 0.00% | 0.00% | 0.00% | 10.29% |
| UK | Weekly | **31.62%** | **24.26%** | **40.44%** | 2.21% |
|  | Fortnightly | 21.32% | 17.65% | 26.47% | 2.21% |
|  | Monthly | 18.38% | 26.47% | 17.65% | 4.41% |
|  | More than once a year | 22.06% | 23.53% | 7.35% | 2.21% |
|  | Yearly | 1.47% | 1.47% | 0.74% | **44.85%** |
|  | Only with a reasonable suspicion | 4.41% | 6.62% | 5.88% | 33.82% |
|  | Never | 0.74% | 0.00% | 1.47% | 10.29% |

**Use of the Bee Health Card**

| Table K. Benefits of using the Bee Health Card. The highest percentages per barrier per country are highlighted in bold. | | | | | | |
| --- | --- | --- | --- | --- | --- | --- |
| Country | **Benefits** | **Agreement** | | | | |
|  |  | **Strongly agree** | **Agree** | **Neutral** | **Disagree** | **Strongly disagree** |
| Estonia | Communication with growers | 9.38% | **50.00%** | 31.25% | 6.25% | 3.13% |
|  | Productivity | 6.25% | **43.75%** | 40.63% | 6.25% | 3.13% |
|  | Quick and easy | 3.13% | 40.63% | **50.00%** | 3.13% | 3.13% |
|  | Lower treatment cost | 3.13% | 40.63% | **43.75%** | 9.38% | 3.13% |
|  | Better crop pollination | 3.13% | 31.25% | **59.38%** | 3.13% | 3.13% |
|  | Increases bee health | 9.38% | **65.63%** | 21.88% | 0.00% | 3.13% |
|  | Environment protection | 3.13% | **43.75%** | 40.63% | 9.38% | 3.13% |
|  | Pollinators protection | 3.13% | **65.63%** | 25.00% | 3.13% | 3.13% |
| Germany | Communication with growers | 9.09% | **36.36%** | 33.33% | 6.06% | 15.15% |
|  | Productivity | 3.03% | 15.15% | **51.52%** | 21.21% | 9.09% |
|  | Quick and easy | 0.00% | **42.42%** | 39.39% | 9.09% | 9.09% |
|  | Lower treatment cost | 0.00% | 15.15% | **60.61%** | 18.18% | 6.06% |
|  | Better crop pollination | 0.00% | 27.27% | **42.42%** | 21.21% | 9.09% |
|  | Increases bee health | 9.09% | **54.55%** | 21.21% | 9.09% | 6.06% |
|  | Environment protection | 6.06% | **42.42%** | 33.33% | 15.15% | 3.03% |
|  | Pollinators protection | 9.09% | **45.45%** | 33.33% | 9.09% | 3.03% |
| Ireland | Communication with growers | 14.78% | 35.65% | **37.39%** | 9.57% | 2.61% |
|  | Productivity | 14.78% | **45.22%** | 33.04% | 5.22% | 1.74% |
|  | Quick and easy | 9.57% | **43.48%** | 41.74% | 4.35% | 0.87% |
|  | Lower treatment cost | 9.57% | 33.04% | **46.09%** | 10.43% | 0.87% |
|  | Better crop pollination | 10.43% | 32.17% | **48.70%** | 7.83% | 0.87% |
|  | Increases bee health | 33.91% | **42.61%** | 21.74% | 0.87% | 0.87% |
|  | Environment protection | 26.09% | 31.30% | **39.13%** | 2.61% | 0.87% |
|  | Pollinators protection | 29.57% | **46.96%** | 20.87% | 1.74% | 0.87% |
| Italy | Communication with growers | 21.21% | **43.94%** | 25.76% | 6.06% | 3.03% |
|  | Productivity | 15.15% | **39.39%** | 33.33% | 12.12% | 0.00% |
|  | Quick and easy | 7.58% | 33.33% | **48.48%** | 10.61% | 0.00% |
|  | Lower treatment cost | 4.55% | 30.30% | **40.91%** | 19.70% | 4.55% |
|  | Better crop pollination | 6.06% | 22.73% | **50.00%** | 18.18% | 3.03% |
|  | Increases bee health | 21.21% | **48.48%** | 19.70% | 7.58% | 3.03% |
|  | Environment protection | 19.70% | **45.45%** | 25.76% | 4.55% | 4.55% |
|  | Pollinators protection | 22.73% | **59.09%** | 15.15% | 1.52% | 1.52% |
| Spain | Communication with growers | 25.00% | **35.00%** | 30.00% | 7.50% | 2.50% |
|  | Productivity | 25.00% | **50.00%** | 15.00% | 7.50% | 2.50% |
|  | Quick and easy | 22.50% | **40.00%** | 32.50% | 2.50% | 2.50% |
|  | Lower treatment cost | 20.00% | **35.00%** | 25.00% | 15.00% | 5.00% |
|  | Better crop pollination | 17.50% | 22.50% | **47.50%** | 7.50% | 5.00% |
|  | Increases bee health | 35.00% | **37.50%** | 20.00% | 5.00% | 2.50% |
|  | Environment protection | 22.50% | **42.50%** | 27.50% | 5.00% | 2.50% |
|  | Pollinators protection | **30.00%** | 40.00% | 22.50% | 5.00% | 2.50% |
| Switzerland | Communication with growers | 19.23% | 26.92% | **36.54%** | 13.46% | 3.85% |
|  | Productivity | 3.85% | 13.46% | **46.15%** | 30.77% | 5.77% |
|  | Quick and easy | 5.77% | 34.62% | **38.46%** | 17.31% | 3.85% |
|  | Lower treatment cost | 5.77% | 11.54% | **42.31%** | 32.69% | 7.69% |
|  | Better crop pollination | 3.85% | 17.31% | **48.08%** | 17.31% | 13.46% |
|  | Increases bee health | 13.46% | 28.85% | **38.46%** | 13.46% | 5.77% |
|  | Environment protection | 7.69% | 25.00% | **42.31%** | 11.54% | 13.46% |
|  | Pollinators protection | 15.38% | 28.85% | **42.31%** | 7.69% | 5.77% |
| UK | Communication with growers | 15.44% | 27.21% | **44.85%** | 8.09% | 4.41% |
|  | Productivity | 9.56% | 28.68% | **52.94%** | 5.88% | 2.94% |
|  | Quick and easy | 11.03% | 38.24% | **39.71%** | 9.56% | 1.47% |
|  | Lower treatment cost | 7.35% | 24.26% | **48.53%** | 16.91% | 2.94% |
|  | Better crop pollination | 8.82% | 18.38% | **58.09%** | 11.76% | 2.94% |
|  | Increases bee health | 29.41% | **40.44%** | 22.79% | 5.15% | 2.21% |
|  | Environment protection | 18.38% | 33.09% | **39.71%** | 6.62% | 2.21% |
|  | Pollinators protection | 25.74% | **36.76%** | 29.41% | 5.88% | 2.21% |
|  | | | | | | |
| Table L. Barriers to using the Bee Health Card. The highest percentages per barrier per country are highlighted in bold. | | | | | | |
| Country | **Barriers** | **Agreement** |  |  |  |  |
|  |  | **Strongly agree** | **Agree** | **Neutral** | **Disagree** | **Strongly disagree** |
| Estonia | No communication with growers | 18.75% | **46.88%** | 25.00% | 9.38% | 0.00% |
|  | Cost | 25.81% | **38.71%** | 22.58% | 12.90% | 0.00% |
|  | Effectiveness | 6.25% | 12.50% | **50.00%** | 28.13% | 3.13% |
|  | Time | 6.25% | 15.63% | **40.63%** | 34.38% | 3.13% |
|  | Difficulty | 3.13% | 12.50% | **40.63%** | 31.25% | 12.50% |
|  | No importance in being used | 3.13% | 3.13% | **40.63%** | 31.25% | 21.88% |
| Germany | No communication with growers | 9.09% | **42.42%** | 36.36% | 3.03% | 9.09% |
|  | Cost | 15.63% | **43.75%** | 34.38% | 6.25% | 0.00% |
|  | Effectiveness | 21.21% | 18.18% | **33.33%** | 27.27% | 0.00% |
|  | Time | 3.03% | 21.21% | **45.45%** | 27.27% | 3.03% |
|  | Difficulty | 0.00% | 12.12% | **51.52%** | 33.33% | 3.03% |
|  | No importance in being used | 18.18% | 15.15% | **39.39%** | 18.18% | 9.09% |
| Ireland | No communication with growers | 21.74% | **39.13%** | 31.30% | 6.96% | 0.87% |
|  | Cost | 25.44% | **40.35%** | 26.32% | 7.89% | 0.00% |
|  | Effectiveness | 2.61% | 32.17% | **42.61%** | 19.13% | 3.48% |
|  | Time | 2.61% | 20.00% | **41.74%** | 29.57% | 6.09% |
|  | Difficulty | 0.87% | 11.30% | **42.61%** | 39.13% | 6.09% |
|  | No importance in being used | 3.48% | 12.17% | **43.48%** | 26.96% | 13.91% |
| Italy | No communication with growers | **42.42%** | 34.85% | 15.15% | 6.06% | 1.52% |
|  | Cost | 18.46% | **33.85%** | 32.31% | 15.38% | 0.00% |
|  | Effectiveness | 10.61% | 28.79% | **33.33%** | 22.73% | 4.55% |
|  | Time | 7.58% | 24.24% | **36.36%** | 31.82% | 0.00% |
|  | Difficulty | 6.06% | 18.18% | 34.85% | **39.39%** | 1.52% |
|  | No importance in being used | 4.55% | 9.09% | 28.79% | **37.88%** | 19.70% |
| Spain | No communication with growers | 30.00% | **40.00%** | 20.00% | 5.00% | 5.00% |
|  | Cost | 20.00% | **32.50%** | **32.50%** | 15.00% | 0.00% |
|  | Effectiveness | 12.50% | 17.50% | **35.00%** | 32.50% | 2.50% |
|  | Time | 0.00% | 12.50% | **45.00%** | 27.50% | 15.00% |
|  | Difficulty | 0.00% | 7.50% | **50.00%** | 35.00% | 7.50% |
|  | No importance in being used | 10.00% | 7.50% | **37.50%** | 27.50% | 17.50% |
| Switzerland | No communication with growers | 7.69% | **48.08%** | 30.77% | 7.69% | 5.77% |
|  | Cost | 17.65% | **52.94%** | 27.45% | 1.96% | 0.00% |
|  | Effectiveness | 15.38% | **40.38%** | 28.85% | 11.54% | 3.85% |
|  | Time | 9.62% | **34.62%** | **34.62%** | 19.23% | 1.92% |
|  | Difficulty | 9.62% | 23.08% | **36.54%** | 23.08% | 7.69% |
|  | No importance in being used | 15.38% | 19.23% | **44.23%** | 11.54% | 9.62% |
| UK | No communication with growers | 25.00% | 30.15% | **39.71%** | 2.21% | 2.94% |
|  | Cost | 33.82% | **38.97%** | 19.85% | 7.35% | 0.00% |
|  | Effectiveness | 11.76% | 26.47% | **42.65%** | 16.91% | 2.21% |
|  | Time | 4.41% | 20.59% | **36.03%** | **36.03%** | 2.94% |
|  | Difficulty | 2.21% | 8.09% | **42.65%** | 41.18% | 5.88% |
|  | No importance in being used | 3.68% | 20.59% | **36.03%** | 30.88% | 8.82% |

| Table M. Confidence levels in the effectiveness of the Bee Health Card. The highest percentages per country are highlighted in bold. | | | | | | | | |
| --- | --- | --- | --- | --- | --- | --- | --- | --- |
| Effectiveness | **Estonia** | **Germany** | **Ireland** | **Italy** | **Spain** | **Switzerland** | UK | **Total** |
| Extremely confident | 12.50% | 3.03% | 12.17% | 9.09% | 17.50% | 1.92% | 5.15% | 8.44% |
| Very confident | 18.75% | **33.33%** | 28.70% | **37.88%** | 30.00% | 28.85% | 24.26% | 28.48% |
| Moderately confident | **46.88%** | 30.30% | **42.61%** | **37.88%** | **40.00%** | **48.08%** | **46.32%** | **42.83%** |
| Slightly confident | 18.75% | 24.24% | 10.43% | 9.09% | 5.00% | 15.38% | 11.76% | 12.24% |
| Not confident at all | 3.13% | 9.09% | 6.09% | 6.06% | 7.50% | 5.77% | 12.50% | 8.02% |

| Table N. Percentage of beekeepers who would use the Bee Health Card with and without economic incentives. According to respondents, the presence of economic incentives would not affect beekeepers’ decision of whether or not to use the tool, and despite the limited available description of the tool and its outputs, about the half of respondents in Ireland, UK, Spain, Italy, and Germany stated they would use it even with extra costs, while more than half of Estonian beekeepers would use it only without extra costs. The highest percentages per country are highlighted in bold. | | | | | | | | | | | | | | | | |
| --- | --- | --- | --- | --- | --- | --- | --- | --- | --- | --- | --- | --- | --- | --- | --- | --- |
| Use with incentives | | **Estonia** | | **Germany** | | **Ireland** | | **Italy** | | **Spain** | | **Switzerland** | | **UK** | | **Total** |
| Yes - even with extra costs | | 40.63% | | **48.48%** | | **49.57%** | | **54.55%** | | **55.00%** | | **40.38%** | | **47.79%** | | **48.52%** |
| Yes - only if there were no extra costs to me | | **56.25%** | | 24.24% | | 43.48% | | 39.39% | | 35.00% | | **40.38%** | | 42.65% | | 41.14% |
| No | | 3.13% | | 27.27% | | 6.96% | | 6.06% | | 10.00% | | 19.23% | | 9.56% | | 10.34% |
| Use without incentives | | **Estonia** | | **Germany** | | **Ireland** | | **Italy** | | **Spain** | | **Switzerland** | | **UK** | | **Total** |
| Yes - even with extra costs | | 34.38% | | **45.45%** | | **49.57%** | | **45.45%** | | **55.00%** | | **46.15%** | | **46.32%** | | **46.84%** |
| Yes - only if there were no extra costs to me | | **56.25%** | | 21.21% | | 40.87% | | 42.42% | | 35.00% | | 38.46% | | 42.65% | | 40.51% |
| No | | 9.38% | | 33.33% | | 9.57% | | 12.12% | | 10.00% | | 15.38% | | 11.03% | | 12.66% |
| Table O. Percentage of beekeepers who would use the Bee Health Card at different regularities with/without economic incentives. Here, the presence of economic incentives would indeed affect beekeepers’ decision of how frequent to use the tool. With no incentives, participants who would use it only with a reasonable suspicion increase by approximately 15 percentage points, while there is a significant decrease among those who would use it more frequently; in particular, beekeepers who would opt for a regular use drop from 24.11% to 12.62% if incentives are not expected. The highest percentages per country are highlighted in bold. | | | | | | | | | | | | | | | | |
| Use frequency with incentives | **Estonia** | | **Germany** | | **Ireland** | | **Italy** | | **Spain** | | **Switzerland** | | **UK** | | **Total** | |
| Regularly | 12.90% | | 20.83% | | 27.10% | | 40.98% | | 13.89% | | 23.81% | | 19.67% | | 24.11% | |
| Irregularly | **41.94%** | | **54.17%** | | **50.47%** | | **40.98%** | | **69.44%** | | **45.24%** | | **51.64%** | | **50.12%** | |
| Suspicion only | **41.94%** | | 25.00% | | 22.43% | | 18.03% | | 13.89% | | 30.95% | | 27.87% | | 25.06% | |
| Never | 3.23% | | 0.00% | | 0.00% | | 0.00% | | 2.78% | | 0.00% | | 0.82% | | 0.71% | |
| Use frequency without incentives | **Estonia** | | **Germany** | | **Ireland** | | **Italy** | | **Spain** | | **Switzerland** | | **UK** | | **Total** | |
| Regularly | 10.34% | | 13.64% | | 15.38% | | 15.79% | | 11.11% | | 18.18% | | 7.50% | | 12.62% | |
| Irregularly | 27.59% | | **54.55%** | | **45.19%** | | **47.37%** | | **50.00%** | | 31.82% | | **50.83%** | | **45.39%** | |
| Suspicion only | **58.62%** | | 31.82% | | 37.50% | | 35.09% | | 33.33% | | **50.00%** | | 40.00% | | 40.05% | |
| Never | 3.45% | | 0.00% | | 1.92% | | 1.75% | | 5.56% | | 0.00% | | 1.67% | | 1.94% | |

| Table P. Health issues the Bee Health Card should be able to detect. Respondents who answered this final open-answer question are 220 (46.41%). The three most cited issues are highlighted in bold. | |
| --- | --- |
| Health issues | **N mentions** |
| Acarine | 4 |
| Bacteria/Bacterial infections/Bacterial diseases | 5 |
| Bee health improvements | 1 |
| Black Queen Cell Virus (BQCV) | 1 |
| Chalkbrood | 5 |
| Chilled brood | 1 |
| Chronic Bee Paralysis Virus (CBPV) | 22 |
| Colony Collapse Disorder | 3 |
| Colony winter loss | 1 |
| Deformed Wing Virus (DWV) | 16 |
| Diseases | 31 |
| Diseases and other issues that cannot be detected by visual inspections | 2 |
| Fat body | 1 |
| Foulbroods | **52** |
| Fungal infections | 2 |
| Genetics | 3 |
| Gut diseases | 1 |
| Honey quality | 1 |
| Mated queen fertility | 1 |
| *Nosema apis* | 41 |
| Nutritional issues | 17 |
| Parasitic Mite Syndrome | 1 |
| Pathogens | 4 |
| Pesticides | **47** |
| Pollution | 5 |
| Queen health | 1 |
| Resilience index | 1 |
| Sacbrood virus | 6 |
| Sour brood | 2 |
| Spiroplasma | 1 |
| Stress | 2 |
| Telecom radiations | 1 |
| Tracheal mites | 2 |
| *Varroa destructor* and viruses linked to it | **56** |
| Viruses (unspecified) | 26 |

| Table Q. Beekeepers belonging to each cluster. Numbers (N) and percentages (%) of beekeepers agreeing, being neutral, and disagreeing with each cluster on MCA maps. | | | | |
| --- | --- | --- | --- | --- |
| Cluster | **Variables** | **Level** | **N beekeepers** | **% beekeepers** |
| Cluster 1: benefits | All benefits | Disagree  Neutral  Agree | 28  188  258 | 6%  40%  54% |
| Cluster 2: barriers | Time, difficulty, importance | Disagree  Neutral  Agree | 161  248  65 | 34%  52%  14% |
