# Supplementary material for: Beekeepers’ perceptions toward a new omics tool for monitoring bee health in Europe
Source: PLoS One. 2025 Jan 14;20(1):e0316609. doi: 10.1371/journal.pone.0316609 (PMC11731711; doi:10.1371/journal.pone.0316609)
Supplement: S4 Appendix — (DOCX) [file pone.0316609.s004.docx]

**Supplementary materials: Beekeepers’ perceptions toward a new omics tool for monitoring bee health in Europe**

Elena Cini^1,2^*, Simon G. Potts^1^, Deepa Senapathi^1^, Matthias Albrecht^3^, Karim Arafah^4^, Dalel Askri^4^, Michel Bocquet^5^, Philippe Bulet^6^, Cecilia Costa^7^, Pilar De la Rúa^8^, Alexandra-Maria Klein^9^, Anina Knauer^3^, Marika Mänd^10^, Risto Raimets^10^, Oliver Schweiger^11,12^, Jane C. Stout^13^, Tom D. Breeze^1^*

^1^Centre for Agri-Environmental Research, School of Agriculture, Policy and Development, University of Reading, Reading, England, United Kingdom

^2^School of Environmental and Natural Sciences, Bangor University, Bangor, Wales, United Kingdom

^3^Agroecology and Environment, Agroscope, Zurich, Switzerland

^4^Plateforme BioPark d’Archamps, Archamps, France

^5^Apimedia, Pringy, Annecy, France

^6^Institute for Advanced Biosciences, CR Inserm U1209, CNRS UMR5309, Université Grenoble Alpes. Team-Verdel: ARN, Epigénétique et Stress/RNA, Epigenetics and Stress, Grenoble, France

^7^CREA Research Centre for Agriculture and Environment, Bologna, Italy

^8^Department of Zoology and Physical Anthropology, Faculty of Veterinary, University of Murcia, Murcia, Spain

^9^Chair of Nature Conservation and Landscape Ecology, University of Freiburg, Freiburg, Germany

^10^Institute of Agricultural and Environmental Sciences, Estonian University of Life Sciences, Tartu, Estonia

^11^UFZ – Helmholtz Centre for Environmental Research, Department of Community Ecology, Halle, Germany

^12^German Centre for Integrative Biodiversity Research (iDiv) Halle-Jena-Leipzig, Deutscher, Leipzig, Germany

^13^Trinity College Dublin, School of Natural Sciences, Botany Department, College Green, Dublin, Ireland

*Corresponding authors

Emails: [elena.cini.ec@gmail.com](mailto:elena.cini.ec@gmail.com) (EC), [t.d.breeze@reading.ac.uk](mailto:t.d.breeze@reading.ac.uk) (TB)

**S4 Appendix. Model selection**

| Table A. Global models before and after removing terms with a Variance Inflation Factor (VIF) ≥5. | | |
| --- | --- | --- |
| Response variable | **Global model before removing terms with VIF ≥5** | **Global model after removing terms with VIF ≥5** |
| Willingness to use the BHC with incentives | Country  bhc.e  bhc.be.p  bhc.be.tc  bhc.ba.c  bhc.ba.t.e.d.i  bhc.be.pp.ep.qe  bhc.be.g.bh.cp | bhc.e  bhc.be.p  bhc.be.tc  bhc.ba.c  bhc.ba.t.e.d.i  bhc.be.pp.ep.qe  bhc.be.g.bh.cp |
| Willingness to use the BHC without incentives | Country  bhc.e  bhc.be.p  bhc.be.tc  bhc.ba.c  bhc.ba.t.e.d.i  bhc.be.pp.ep.qe  bhc.be.g.bh.cp | Country  bhc.e  bhc.be.p  bhc.be.tc  bhc.ba.t.e.d.i  bhc.be.pp.ep.qe  bhc.be.g.bh.cp |
| Willingness to accept BHC extra costs with incentives | Country  bhc.e  bhc.be.p  bhc.be.tc  bhc.ba.c  bhc.ba.t.e.d.i  bhc.be.pp.ep.qe  bhc.be.g.bh.cp | Country  bhc.e  bhc.be.p  bhc.be.tc  bhc.ba.c  bhc.ba.t.e.d.i |
| Willingness to accept BHC extra costs without incentives | Country  bhc.e  bhc.be.p  bhc.be.tc  bhc.ba.c  bhc.ba.t.e.d.i  bhc.be.pp.ep.qe  bhc.be.g.bh.cp | Country  bhc.e  bhc.be.p  bhc.be.tc  bhc.ba.c  bhc.ba.t.e.d.i |
| Frequency of BHC use with incentives | Country  bhc.e  bhc.be.p  bhc.be.tc  bhc.ba.c  bhc.ba.t.e.d.i  bhc.be.pp.ep.qe  bhc.be.g.bh.cp | Country  bhc.e  bhc.be.p  bhc.be.tc  bhc.ba.c  bhc.ba.t.e.d.i |
| Frequency of BHC use without incentives | Country  bhc.e  bhc.be.p  bhc.be.tc  bhc.ba.c  bhc.ba.t.e.d.i  bhc.be.pp.ep.qe  bhc.be.g.bh.cp | Country  bhc.e  bhc.be.p  bhc.be.tc  bhc.ba.c  bhc.ba.t.e.d.i |

| Table B. Backward stepwise model selection from global models (including all terms with VIF <5) to final models (including only significant terms, with p-value <0.05). Final models are highlighted in bold. |
| --- |
| Willingness to use the BHC with economic incentives |
| Terms |
| (5) bhc.e + bhc.be.p + bhc.ba.t.e.d.i |
| (4) bhc.e + bhc.be.p + bhc.ba.c + bhc.ba.t.e.d.i |
| (3) bhc.e + bhc.be.p + bhc.ba.c + bhc.ba.t.e.d.i + bhc.be.pp.ep.qe |
| (2) bhc.e + bhc.be.p + bhc.be.tc + bhc.ba.c + bhc.ba.t.e.d.i + bhc.be.pp.ep.qe |
| (1) bhc.e + bhc.be.p + bhc.be.tc + bhc.ba.c + bhc.ba.t.e.d.i + bhc.be.pp.ep.qe + bhc.be.g.bh.cp |
| Willingness to use the BHC without economic incentives |
| Terms |
| (5) bhc.e + bhc.ba.t.e.d.i + bhc.be.pp.ep.qe |
| (4) Country + bhc.e + bhc.ba.t.e.d.i + bhc.be.pp.ep.qe |
| (3) Country + bhc.e + bhc.be.tc + bhc.ba.t.e.d.i + bhc.be.pp.ep.qe |
| (2) Country + bhc.e + bhc.be.p + bhc.be.tc + bhc.ba.t.e.d.i + bhc.be.pp.ep.qe |
| (1) Country + bhc.e + bhc.be.p + bhc.be.tc + bhc.ba.t.e.d.i + bhc.be.pp.ep.qe + bhc.be.g.bh.cp |
| Willingness to accept BHC extra costs with economic incentives |
| Terms |
| (5) bhc.e + bhc.ba.t.e.d.i |
| (4) bhc.e + bhc.be.p + bhc.ba.t.e.d.i |
| (3) bhc.e + bhc.be.p + bhc.ba.c + bhc.ba.t.e.d.i |
| (2) bhc.e + bhc.be.p + bhc.be.tc + bhc.ba.c + bhc.ba.t.e.d.i |
| (1) Country + bhc.e + bhc.be.p + bhc.be.tc + bhc.ba.c + bhc.ba.t.e.d.i |
| Willingness to accept BHC extra costs without economic incentives |
| (4) bhc.e + bhc.ba.c + bhc.ba.t.e.d.i |
| (3) bhc.e + bhc.be.p + bhc.ba.c + bhc.ba.t.e.d.i |
| (2) bhc.e + bhc.be.p + bhc.be.tc + bhc.ba.c + bhc.ba.t.e.d.i |
| (1) Country + bhc.e + bhc.be.p + bhc.be.tc + bhc.ba.c + bhc.ba.t.e.d.i |
| Frequency of BHC use with economic incentives |
| Terms |
| (5) bhc.e + bhc.ba.c |
| (4) bhc.e + bhc.be.p + bhc.ba.c |
| (3) bhc.e + bhc.be.p + bhc.ba.c + bhc.ba.t.e.d.i |
| (2) Country + bhc.e + bhc.be.p + bhc.ba.c + bhc.ba.t.e.d.i |
| (1) Country + bhc.e + bhc.be.p + bhc.be.tc + bhc.ba.c + bhc.ba.t.e.d.i |
| Frequency of BHC use without economic incentives |
| Terms |
| (5) bhc.e + bhc.ba.c |
| (4) Country + bhc.e + bhc.ba.c |
| (3) Country + bhc.e + bhc.be.p + bhc.ba.c |
| (2) Country + bhc.e + bhc.be.p + bhc.be.tc + bhc.ba.c |
| (1) Country + bhc.e + bhc.be.p + bhc.be.tc + bhc.ba.c + bhc.ba.t.e.d.i |
