# Supplementary material for: Beekeepers’ perceptions toward a new omics tool for monitoring bee health in Europe
Source: PLoS One. 2025 Jan 14;20(1):e0316609. doi: 10.1371/journal.pone.0316609 (PMC11731711; doi:10.1371/journal.pone.0316609)
Supplement: S5 Appendix — (DOCX) [file pone.0316609.s005.docx]

**Supplementary materials: Beekeepers’ perceptions toward a new omics tool for monitoring bee health in Europe**

Elena Cini^1,2^*, Simon G. Potts^1^, Deepa Senapathi^1^, Matthias Albrecht^3^, Karim Arafah^4^, Dalel Askri^4^, Michel Bocquet^5^, Philippe Bulet^6^, Cecilia Costa^7^, Pilar De la Rúa^8^, Alexandra-Maria Klein^9^, Anina Knauer^3^, Marika Mänd^10^, Risto Raimets^10^, Oliver Schweiger^11,12^, Jane C. Stout^13^, Tom D. Breeze^1^*

^1^Centre for Agri-Environmental Research, School of Agriculture, Policy and Development, University of Reading, Reading, England, United Kingdom

^2^School of Environmental and Natural Sciences, Bangor University, Bangor, Wales, United Kingdom

^3^Agroecology and Environment, Agroscope, Zurich, Switzerland

^4^Plateforme BioPark d’Archamps, Archamps, France

^5^Apimedia, Pringy, Annecy, France

^6^Institute for Advanced Biosciences, CR Inserm U1209, CNRS UMR5309, Université Grenoble Alpes. Team-Verdel: ARN, Epigénétique et Stress/RNA, Epigenetics and Stress, Grenoble, France

^7^CREA Research Centre for Agriculture and Environment, Bologna, Italy

^8^Department of Zoology and Physical Anthropology, Faculty of Veterinary, University of Murcia, Murcia, Spain

^9^Chair of Nature Conservation and Landscape Ecology, University of Freiburg, Freiburg, Germany

^10^Institute of Agricultural and Environmental Sciences, Estonian University of Life Sciences, Tartu, Estonia

^11^UFZ – Helmholtz Centre for Environmental Research, Department of Community Ecology, Halle, Germany

^12^German Centre for Integrative Biodiversity Research (iDiv) Halle-Jena-Leipzig, Deutscher, Leipzig, Germany

^13^Trinity College Dublin, School of Natural Sciences, Botany Department, College Green, Dublin, Ireland

*Corresponding authors

Emails: [elena.cini.ec@gmail.com](mailto:elena.cini.ec@gmail.com) (EC), [t.d.breeze@reading.ac.uk](mailto:t.d.breeze@reading.ac.uk) (TB)

**S5 Appendix. Regression model results and coefficients**

| Table A. Final model investigating the willingness to use the BHC with economic incentives. Goodness-of-fit > 0.05 indicates no evidence of a lack of model fit. Significant p-values (>0.05) are highlighted in bold. | | | |
| --- | --- | --- | --- |
| Willingness to use the PoshBee tool with economic incentives | | | |
| Terms | **χ^2^** | **df** | **p-value** |
| Confidence level in effectiveness | 19.72 | 2 | **<0.001** |
| Productivity as benefit | 11.79 | 2 | **0.003** |
| Time, effectiveness, difficulty, and importance as barriers | 11.26 | 2 | **0.004** |
| Goodness-of-fit | **χ^2^** | **df** | **p-value** |
| Hosmer-Lemeshow test | 5.64 | 5 | 0.343 |
| Model summary | **R^2^**  22.21 |  |  |

| Table B. Final model investigating the willingness to accept BHC extra costs with economic incentives. Goodness-of-fit > 0.05 indicates no evidence of a lack of model fit. Significant p-values (>0.05) are highlighted in bold. | | | |
| --- | --- | --- | --- |
| Willingness to accept extra costs related to the PoshBee tool with economic incentives | | | |
| Terms | **χ^2^** | **df** | **p-value** |
| Confidence level in effectiveness | 19.47 | 2 | **<0.001** |
| Time, effectiveness, difficulty, and importance as barriers | 25.81 | 2 | **<0.001** |
| Goodness-of-fit | **χ^2^** | **df** | **p-value** |
| Hosmer-Lemeshow test | 1.37 | 2 | 0.503 |
| Model summary | **R^2^**  10.99 |  |  |

| Table C. Final model investigating the willingness to use the BHC without economic incentives. Goodness-of-fit > 0.05 indicates no evidence of a lack of model fit. Significant p-values (>0.05) are highlighted in bold. | | | | | | |
| --- | --- | --- | --- | --- | --- | --- |
| Willingness to use the PoshBee tool without economic incentives | | | | | | |
| Terms | | **χ^2^** | | **df** | **p-value** | |
| Confidence level in effectiveness | | 18.46 | | 2 | **<0.001** | |
| Time, effectiveness, difficulty, and importance as barriers | | 7.13 | | 2 | **0.028** | |
| Pollinator protection, environment protection, and easy to use as benefits | | 15.95 | | 2 | **<0.001** | |
| Goodness-of-fit | | **χ^2^** | | **df** | **p-value** | |
| Hosmer-Lemeshow test | | 0.59 | | 2 | 0.965 | |
| Model summary | | **R^2^**  24.80 | |  |  | |
| Table D. Final model investigating the willingness to accept BHC extra costs without economic incentives. Goodness-of-fit > 0.05 indicates no evidence of a lack of model fit. Significant p-values (>0.05) are highlighted in bold. | | | | | |  |
| Willingness to accept extra costs related to the PoshBee tool without economic incentives | | | | | |  |
| Terms | **χ^2^** | | **df** | | **p-value** |  |
| Confidence level in effectiveness | 15.11 | | 2 | | **0.001** |  |
| Cost as barrier | 8.37 | | 2 | | **0.015** |  |
| Time, effectiveness, difficulty, and importance as barriers | 14.03 | | 2 | | **0.001** |  |
| Goodness-of-fit | **χ^2^** | | **df** | | **p-value** |  |
| Hosmer-Lemeshow test | 3.39 | | 5 | | 0.640 |  |
| Model summary | **R^2^**  10.33 | |  | |  |  |

| Table E. Final model investigating the BHC use frequency with economic incentives. Goodness-of-fit > 0.05 indicates no evidence of a lack of model fit. Significant p-values (>0.05) are highlighted in bold. | | | |
| --- | --- | --- | --- |
| Frequency of use of the PoshBee tool with economic incentives | | | |
| Terms | **χ^2^** | **df** | **p-value** |
| Confidence level in effectiveness | 20.81 | 2 | **<0.001** |
| Cost as barrier | 6.53 | 2 | **0.038** |
| Goodness-of-fit | **χ^2^** | **df** | **p-value** |
| Hosmer-Lemeshow test | 2.52 | 2 | 0.283 |
| Model summary | **R^2^**  6.60 |  |  |

| Table F. Final model investigating the BHC use frequency without economic incentives. Goodness-of-fit > 0.05 indicates no evidence of a lack of model fit. Significant p-values (>0.05) are highlighted in bold. | | | |
| --- | --- | --- | --- |
| Frequency of use of the PoshBee tool without economic incentives | | | |
| Terms | **χ^2^** | **df** | **p-value** |
| Confidence level in effectiveness | 23.42 | 2 | **<0.001** |
| Cost as barrier | 13.54 | 2 | **0.001** |
| Goodness-of-fit | **χ^2^** | **df** | **p-value** |
| Hosmer-Lemeshow test | 1.01 | 3 | 0.798 |
| Model summary | **R^2^**  7.92 |  |  |

| Table G. Coefficients, SE, z-values, p-values, and VIFs of final model terms. Variables used as reference values are ‘fair confidence’ and ‘neutral’ answers. Significant p-values (<0.050) are highlighted in bold and indicate significant differences from the reference variables. | | | | | |
| --- | --- | --- | --- | --- | --- |
| Willingness to use the BHC with incentives | **Coefficient values** | | | | |
| Term | **Coeff** | **SE** | **Z-Value** | **P-Value** | **VIF** |
| Confidence level in effectiveness  High confidence  No confidence | 0.706  -1.630 | 0.536  0.418 | 1.32  -3.90 | 0.188  **<0.001** | 1.19  1.09 |
| Productivity as benefit  Disagree  Agree | -0.791  0.963 | 0.391  0.468 | -2.03  2.06 | **0.043**  **0.040** | 1.14  1.17 |
| Time, effectiveness, difficulty, and importance as barriers  Disagree  Agree | 0.065  -1.163 | 0.563  0.366 | 0.11  -3.18 | 0.908  **0.001** | 1.19  1.16 |
| Willingness to use the BHC without incentives | **Coefficient values** | | | | |
| Term | **Coeff** | **SE** | **Z-Value** | **P-Value** | **VIF** |
| Confidence level in effectiveness  High confidence  No confidence | 1.535  -1.366 | 0.651  0.413 | 2.36  -3.31 | **0.018**  **0.001** | 1.15  1.07 |
| Time, effectiveness, difficulty, and importance as barriers  Disagree  Agree | -0.313  -0.918 | 0.492  0.345 | -0.64  -2.66 | 0.524  **0.008** | 1.18  1.16 |
| Pollinator protection, environment protection, and easy to use the tool as benefits  Disagree  Agree | -1.097  1.058 | 0.466  0.393 | -2.36  2.69 | **0.018**  **0.007** | 1.11  1.17 |
| Willingness to accept BHC extra costs with incentives | **Coefficient values** | | | | |
| Term | **Coeff** | **SE** | **Z-Value** | **P-Value** | **VIF** |
| Confidence level in effectiveness  High confidence  No confidence | 0.475  -1.902 | 0.215  0.552 | 2.21  -3.45 | **0.027**  **0.001** | 1.02 |
| Time, effectiveness, difficulty, and importance as barriers  Disagree  Agree | 0.778  -0.894 | 0.255  0.268 | 3.05  -3.34 | **0.002**  **0.001** | 1.10  1.09 |
| Willingness to accept BHC extra costs without incentives | **Coefficient values** | | | | |
| Term | **Coeff** | **SE** | **Z-Value** | **P-Value** | **VIF** |
| Confidence level in effectiveness  High confidence  No confidence | 0.306  -1.866 | 0.216  0.555 | 1.42  -3.36 | 0.156  **0.001** | 1.12  1.02 |
| Cost as barrier  Disagree  Agree | 0.624  -0.381 | 0.403  0.227 | 1.55  -1.68 | 0.122  **0.094** | 1.23  1.21 |
| Time, effectiveness, difficulty, and importance as barriers  Disagree  Agree | 0.655  -0.593 | 0.257  0.267 | 2.54  -2.22 | **0.011**  **0.026** | 1.15  1.13 |
| Frequency of use of the BHC with incentives | **Coefficient values** | | | | |
| Term | **Coeff** | **SE** | **Z-Value** | **P-Value** | **VIF** |
| Confidence level in effectiveness  High confidence  No confidence | 0.837  -1.262 | 0.260  0.476 | 3.22  -2.65 | **0.001**  **0.008** | 1.04  1.03 |
| Cost as barrier  Disagree  Agree | -0.310  -0.734 | 0.482  0.296 | -0.64  -2.48 | 0.520  **0.013** | 1.33  1.31 |
| Frequency of use of the BHC without incentives | **Coefficient values** | | | | |
| Term | **Coeff** | **SE** | **Z-Value** | **P-Value** | **VIF** |
| Confidence level in effectiveness  High confidence  No confidence | 0.877  -1.273 | 0.221  0.586 | 3.96  -2.17 | **<0.001**  **0.030** | 1.03  1.02 |
| Cost as barrier  Disagree  Agree | 0.287  -0.742 | 0.428  0.249 | 0.67  -2.98 | 0.503  **0.003** | 1.23  1.23 |
