# Supplementary material for: Beekeepers’ perceptions toward a new omics tool for monitoring bee health in Europe
Source: PLoS One. 2025 Jan 14;20(1):e0316609. doi: 10.1371/journal.pone.0316609 (PMC11731711; doi:10.1371/journal.pone.0316609)
Supplement: S6 Appendix — (DOCX) [file pone.0316609.s006.docx]

**Supplementary materials: Beekeepers’ perceptions toward a new omics tool for monitoring bee health in Europe**

Elena Cini^1,2^*, Simon G. Potts^1^, Deepa Senapathi^1^, Matthias Albrecht^3^, Karim Arafah^4^, Dalel Askri^4^, Michel Bocquet^5^, Philippe Bulet^6^, Cecilia Costa^7^, Pilar De la Rúa^8^, Alexandra-Maria Klein^9^, Anina Knauer^3^, Marika Mänd^10^, Risto Raimets^10^, Oliver Schweiger^11,12^, Jane C. Stout^13^, Tom D. Breeze^1^*

^1^Centre for Agri-Environmental Research, School of Agriculture, Policy and Development, University of Reading, Reading, England, United Kingdom

^2^School of Environmental and Natural Sciences, Bangor University, Bangor, Wales, United Kingdom

^3^Agroecology and Environment, Agroscope, Zurich, Switzerland

^4^Plateforme BioPark d’Archamps, Archamps, France

^5^Apimedia, Pringy, Annecy, France

^6^Institute for Advanced Biosciences, CR Inserm U1209, CNRS UMR5309, Université Grenoble Alpes. Team-Verdel: ARN, Epigénétique et Stress/RNA, Epigenetics and Stress, Grenoble, France

^7^CREA Research Centre for Agriculture and Environment, Bologna, Italy

^8^Department of Zoology and Physical Anthropology, Faculty of Veterinary, University of Murcia, Murcia, Spain

^9^Chair of Nature Conservation and Landscape Ecology, University of Freiburg, Freiburg, Germany

^10^Institute of Agricultural and Environmental Sciences, Estonian University of Life Sciences, Tartu, Estonia

^11^UFZ – Helmholtz Centre for Environmental Research, Department of Community Ecology, Halle, Germany

^12^German Centre for Integrative Biodiversity Research (iDiv) Halle-Jena-Leipzig, Deutscher, Leipzig, Germany

^13^Trinity College Dublin, School of Natural Sciences, Botany Department, College Green, Dublin, Ireland

*Corresponding authors

Emails: [elena.cini.ec@gmail.com](mailto:elena.cini.ec@gmail.com) (EC), [t.d.breeze@reading.ac.uk](mailto:t.d.breeze@reading.ac.uk) (TB)

**S6 Appendix. Cost of using and managing the Bee Health Card**

| Table A. Costs of using the BHC tool per use (assuming 10 samples/use). | |
| --- | --- |
| Cost type | **Cost/use** |
| Beekeeper consumable costs per use | €2.24 |
| Postage costs | €8.95-€32.03 |
| Lab consumable costs per use | €15.54 |
| Staff costs per use | €6.69-€14.97 |
| Data storage per use (~2.5MB) | €0.001 |
| Total costs/use | **€33.83 (ESP) – €45.66 (DEU)** |

| Table B. Total national running costs of the BHC under high rates of adoption. | | | | | | | | | |
| --- | --- | --- | --- | --- | --- | --- | --- | --- | --- |
| Country | **N° beekeepers** | **Adoption rate^1^** | **Samples per year^2^** | **BK fixed costs^3^**  **(€,000)** | **BK variable costs^4^**  **(€,000)** | **Postage costs^5^**  **(€,000)** | **Analytical costs^6^**  **(€,000)** | **Admin costs^7^**  **(€,000)** | **Total costs**  **(€,000)** |
| Estonia | 5,215 | 97% | 50,481 | € 8 | € 4 | € 46 | € 45 | € 29 | € 133 |
| Germany | 116,000 | 73% | 843,320 | € 226 | € 118 | € 1,676 | € 1,443 | € 61 | € 3,535 |
| Ireland | 3,300 | 94% | 30,888 | € 7 | € 4 | € 42 | € 43 | € 53 | € 141 |
| Italy | 56,059 | 94% | 526,955 | € 109 | € 57 | € 518 | € 685 | € 59 | € 1,338 |
| Spain | 28,786 | 90% | 259,074 | € 68 | € 35 | € 298 | € 352 | € 49 | € 802 |
| Switzerland | 18,150 | 81% | 146,652 | € 36 | € 19 | € 182 | € 256 | € 41 | € 534 |
| UK | 39,475 | 90% | 357,173 | € 79 | € 41 | € 164 | € 558 | € 41 | € 883 |
| ^1^Rate of adoption among beekeepers when the BHC is provided with economic incentives and with no extra costs. ^2^Number of samples to be process, assuming each user sends in 10 samples (figures rounded to the nearest 10). ^3^Cost of reusable materials each beekeeper must use. ^4^Costs of materials that are consumed with each use of the health card. ^5^Costs of postage using half standard international carrier rates. ^6^Costs associated with lab work per sample analysed. ^7^Salary of an administrator. | | | | | | | | | |

| Table C. Total national running costs of the BHC under low rates of adoption. | | | | | | | | | |
| --- | --- | --- | --- | --- | --- | --- | --- | --- | --- |
| Country | **N° beekeepers** | **Adoption rate^1^** | **Samples per year^2^** | **BK fixed costs^3^ (€,000)** | **BK variable costs^4^ (€,000)** | **Postage costs^5^ (€,000)** | **Analytical costs^6^ (€,000)** | **Admin costs^7^ (€,000)** | **Total costs (€,000)** |
| Estonia | 5,215 | 34% | 50,480 | € 8 | € 4 | € 47 | € 28 | € 29 | € 115 |
| Germany | 116,000 | 45% | 843,320 | € 226 | € 118 | € 1,687 | € 818 | € 61 | € 2,909 |
| Ireland | 3,300 | 50% | 30,890 | € 7 | € 4 | € 42 | € 25 | € 53 | € 131 |
| Italy | 56,059 | 45% | 526,960 | € 109 | € 57 | € 517 | € 395 | € 59 | € 1,138 |
| Spain | 28,786 | 55% | 259,070 | € 68 | € 35 | € 297 | € 246 | € 49 | € 694 |
| Switzerland | 18,150 | 46% | 146,650 | € 36 | € 19 | € 183 | € 130 | € 41 | € 408 |
| UK | 39,475 | 46% | 357,170 | € 79 | € 41 | € 164 | € 284 | € 41 | € 609 |
| ^1^Rate of adoption among beekeepers when the BHC is provided with no economic incentives and with extra costs. ^2^Number of samples to be process, assuming each user sends in 10 samples (figures rounded to the nearest 10). ^3^Cost of reusable materials each beekeeper must use. ^4^Costs of materials that are consumed with each use of the health card. ^5^Costs of postage using half standard international carrier rates. ^6^Costs associated with lab work per sample analysed. ^7^Salary of an administrator. | | | | | | | | | |

| Table D. Projected impacts on winter colony losses under a pessimistic efficiency frontier. | | | | | | | | | |
| --- | --- | --- | --- | --- | --- | --- | --- | --- | --- |
|  |  |  |  |  |  | **% Increase in survival^6^** | | **Extra colonies surviving^7^** | |
| Country | **Total colonies^1^** | **Winter loss^2^** | **Losses^3^ (status Quo)** | **Adoption (high)^4^** | **Adoption (low)^5^** | **High Ad** | **Low Ad** | **High Ad** | **Low Ad** |
| Estonia | 48,720 | 8.30% | 4,044 | 97% | 34% | 47% | 6% | 1,895 | 239 |
| Germany | 771,850 | 11.60% | 89,535 | 73% | 45% | 26% | 10% | 23,661 | 9,227 |
| Ireland | 22278 | 3.90% | 869 | 94% | 50% | 44% | 12% | 381 | 107 |
| Italy | 423,144 | 8.80% | 37,237 | 94% | 45% | 44% | 10% | 16,451 | 3,838 |
| Spain | 2,901,680 | 17.60% | 510,696 | 90% | 55% | 41% | 15% | 206,832 | 77,243 |
| Switzerland | 179,473 | 7.40% | 13,281 | 81% | 46% | 33% | 11% | 4,335 | 1,417 |
| UK | 255,000 | 5.40% | 13,770 | 90% | 46% | 41% | 11% | 5,636 | 1,476 |
| ^1^Total estimated colony numbers from FAOSTAT, 2022, NBU, 2022 (GBR) and EC, 2021c (IRE). ^2^Percentage of winter colony losses as reported in Gray et al., 2020. ^3^Number of colonies projected to be lost with no intervention. ^4^Rate of adoption among beekeepers when the BHC is provided with economic incentives and with no extra costs. ^5^Rate of adoption among beekeepers when the BHC is provided with no economic incentives and with extra costs. ^6^Percentage of reduction in colony losses thanks to the BHC, based on a maximum 50% with total adoption. ^7^Number of colonies that survive thanks to the BHC. | | | | | | | | | |

| Table E. Projected impacts on winter colony losses under a linear efficiency frontier. | | | | | | | | | |
| --- | --- | --- | --- | --- | --- | --- | --- | --- | --- |
|  |  |  |  |  |  | **% Increase in survival^6^** | | **Extra colonies surviving^7^** | |
| Country | **Total colonies^1^** | **Winter loss^2^** | **Losses (status Quo)^3^** | **Adoption (high)^4^** | **Adoption (low)^5^** | **High Ad** | **Low Ad** | **High Ad** | **Low Ad** |
| Estonia | 48,720 | 8.30% | 4,044 | 97% | 34% | 50% | 28% | 2,020 | 1,152 |
| Germany | 771,850 | 11.60% | 89,535 | 73% | 45% | 46% | 35% | 41,431 | 31,421 |
| Ireland | 22278 | 3.90% | 869 | 94% | 50% | 50% | 37% | 433 | 324 |
| Italy | 423,144 | 8.80% | 37,237 | 94% | 45% | 50% | 35% | 18,551 | 13,068 |
| Spain | 2,901,680 | 17.60% | 510,696 | 90% | 55% | 50% | 40% | 252,794 | 203,640 |
| Switzerland | 179,473 | 7.40% | 13,281 | 81% | 46% | 48% | 36% | 6,396 | 4,718 |
| UK | 255,000 | 5.40% | 13,770 | 90% | 46% | 50% | 36% | 6,823 | 4,900 |
| ^1^Total estimated colony numbers from FAOSTAT, 2022, NBU, 2022 (GBR) and EC, 2021c (IRE). ^2^Percentage of winter colony losses as reported in Gray et al., 2020. ^3^Number of colonies projected to be lost with no intervention. ^4^Rate of adoption among beekeepers when the BHC is provided with economic incentives and with no extra costs. ^5^Rate of adoption among beekeepers when the BHC is provided with no economic incentives and with extra costs. ^6^Percentage of reduction in colony losses thanks to the BHC, based on a maximum 50% with total adoption. ^7^Number of colonies that survive thanks to the BHC. | | | | | | | | | |

| Table F. Projected impacts on winter colony losses under the optimistic efficiency frontier. | | | | | | | | | |
| --- | --- | --- | --- | --- | --- | --- | --- | --- | --- |
|  |  |  |  |  |  | **% Increase in survival^6^** | | **Extra colonies surviving^7^** | |
| Country | **Total colonies^1^** | **Winter loss^2^** | **Losses (status Quo)^3^** | **Adoption (high)^4^** | **Adoption (low)^5^** | **High Ad** | **Low Ad** | **High Ad** | **Low Ad** |
| Estonia | 48,720 | 8.30% | 4,044 | 97% | 34% | 97% | 34% | 3,914 | 1,391 |
| Germany | 771,850 | 11.60% | 89,535 | 73% | 45% | 73% | 45% | 65,092 | 40,649 |
| Ireland | 22278 | 3.90% | 869 | 94% | 50% | 94% | 50% | 813 | 431 |
| Italy | 423,144 | 8.80% | 37,237 | 94% | 45% | 94% | 45% | 35,002 | 16,905 |
| Spain | 2,901,680 | 17.60% | 510,696 | 90% | 55% | 90% | 55% | 459,626 | 280,883 |
| Switzerland | 179,473 | 7.40% | 13,281 | 81% | 46% | 81% | 46% | 10,731 | 6,136 |
| UK | 255,000 | 5.40% | 13,770 | 90% | 46% | 90% | 46% | 12,459 | 6,376 |
| ^1^Total estimated colony numbers from FAOSTAT, 2022, NBU, 2022 (GBR) and EC, 2021c (IRE). ^2^Percentage of winter colony losses as reported in Gray et al., 2020. ^3^Number of colonies projected to be lost with no intervention. ^4^Rate of adoption among beekeepers when the BHC is provided with economic incentives and with no extra costs. ^5^Rate of adoption among beekeepers when the BHC is provided with no economic incentives and with extra costs. ^6^Percentage of reduction in colony losses thanks to the BHC, based on a maximum 50% with total adoption. ^7^Number of colonies that survive thanks to the BHC. | | | | | | | | | |
