# Supplementary material for: Beekeepers’ perceptions toward a new omics tool for monitoring bee health in Europe
Source: PLoS One. 2025 Jan 14;20(1):e0316609. doi: 10.1371/journal.pone.0316609 (PMC11731711; doi:10.1371/journal.pone.0316609)
Supplement: Attachment — Submitted filename: Response to PLOS ONE Editor re copyright claims.docx [file pone.0316609.s007.docx]

**Response to copyright claims of Fig. 1**

Dear Editorial Manager,

You have stated that Fig. 1 in our submission contains copyrighted images.

However, this is not true. All images have been taken from Clipsafari ([www.clipsafari.com](http://www.clipsafari.com)) and have no copyright. Under each figure on the website, it is reported the following: “Image License: CC0 No Rights Reserved. You can copy, modify, distribute and perform the work, even for commercial purposes, all without asking permission, and at no cost. This picture is completely free.”

Please refer to websites and the screenshots below, and please do let us know if you have further questions.

Websites: <https://www.clipsafari.com/clips/o253309-scientist> ; <https://www.clipsafari.com/clips/o306649-report> ; <https://www.clipsafari.com/clips/o309882-bees-and-hive> ; <https://www.clipsafari.com/clips/o283154-beekeeper-avatar>

Screenshots from websites:
